# Systematic characterization of gene families and functional analysis of *PvRAS3* and *PvRAS4* involved in rosmarinic acid biosynthesis in *Prunella vulgaris*

**DOI:** 10.3389/fpls.2024.1374912

**Published:** 2024-05-01

**Authors:** Chao Yan, Caili Li, Maochang Jiang, Yayun Xu, Sixuan Zhang, Xiangling Hu, Yuhang Chen, Shanfa Lu

**Affiliations:** ^1^ State Key Laboratory for Quality Ensurance and Sustainable Use of Dao-di Herbs, Institute of Medicinal Plant Development, Chinese Academy of Medical Sciences & Peking Union Medical College, Beijing, China; ^2^ Engineering Research Center of Chinese Medicine Resource, Ministry of Education, Institute of Medicinal Plant Development, Chinese Academy of Medical Sciences & Peking Union Medical College, Beijing, China; ^3^ College of Pharmaceutical Sciences, Chengdu Medical College, Chengdu, China

**Keywords:** biosynthetic pathway, CRISPR/Cas9, *in vitro* enzymatic activity assay, *Prunella vulgaris*, PvRAS3, PvRAS4, rosmarinic acid, rosmarinic acid synthase

## Abstract

*Prunella vulgaris* is an important material for Chinese medicines with rosmarinic acid (RA) as its index component. Based on the chromosome-level genome assembly we obtained recently, 51 RA biosynthesis-related genes were identified. Sequence feature, gene expression pattern and phylogenetic relationship analyses showed that 17 of them could be involved in RA biosynthesis. *In vitro* enzymatic assay showed that PvRAS3 catalyzed the condensation of *p*-coumaroyl-CoA and caffeoyl-CoA with pHPL and DHPL. Its affinity toward *p*-coumaroyl-CoA was higher than caffeoyl-CoA. PvRAS4 catalyzed the condensation of *p*-coumaroyl-CoA with pHPL and DHPL. Its affinity toward *p*-coumaroyl-CoA was lower than PvRAS3. UPLC and LC-MS/MS analyses showed the existence of RA, 4-coumaroyl-3’,4’-dihydroxyphenyllactic acid, 4-coumaroyl-4’-hydroxyphenyllactic acid and caffeoyl-4’-hydroxyphenyllactic acid in *P. vulgaris*. Generation and analysis of *pvras3* homozygous mutants showed significant decrease of RA, 4-coumaroyl-3’,4’-dihydroxyphenyllactic acid, 4-coumaroyl-4’-hydroxyphenyllactic acid and caffeoyl-4’-hydroxyphenyllactic acid and significant increase of DHPL and pHPL. It suggests that PvRAS3 is the main enzyme catalyzing the condensation of acyl donors and acceptors during RA biosynthesis. The role of PvRAS4 appears minor. The results provide significant information for quality control of *P. vulgaris* medicinal materials.

## Introduction

*Prunella vulgaris* L. is a perennial medicinal plant of Lamiaceae, which is widely distributed in Asia, North America, Europe and North Africa (National Pharmacopoeia Committee, 2020; Hu et al., 2023). The whole plants and spikes of *P. vulgaris* are commonly used to treat thyroiditis, mastitis, tuberculosis, infectious hepatitis and hypertension in East Asia, the Middle East, and Europe (Tang et al., 2023). In addition, *P. vulgaris* spikes are used as the main raw materials of functional herbal tea in the southern provinces of China. Its fresh leaves are used as seasonal vegetables in southeastern China. The whole plants are often used as urban landscape plants for urban greening (Chen et al., 2019). The demand for *P. vulgaris* in the production of Chinese patented medicines and functional herbal tea is approximately 60 million kilograms per year (Chen et al., 2022; Li et al., 2022).

*P. vulgaris* is rich in polyphenols, of which rosmarinic acid (RA) is an index component in evaluating the quality of *P. vulgaris* medicinal materials and Chinese patented medicines. As the main polyphenol component produced in *P. vulgaris*, RA has a variety of pharmacological activities, such as antioxidant, anti-inflammatory, anti-tumor, anti-allergy, anti-depression, and anti-anxiety (Taguchi et al., 2017). It also has unique pharmacological effects in improving sleep, neurological prevention, reducing testicular injury and inhibiting elastin degradation (Kwon et al., 2017), and has obvious inhibitory effect on liver tumor cells, lung tumor cells and stomach tumor cells (Radziejewska et al., 2018). In addition, RA is easily absorbed and no toxic side effects on blood cells, kidney, and liver (Noguchi-Shinohara et al., 2015).

**Figure 1:**
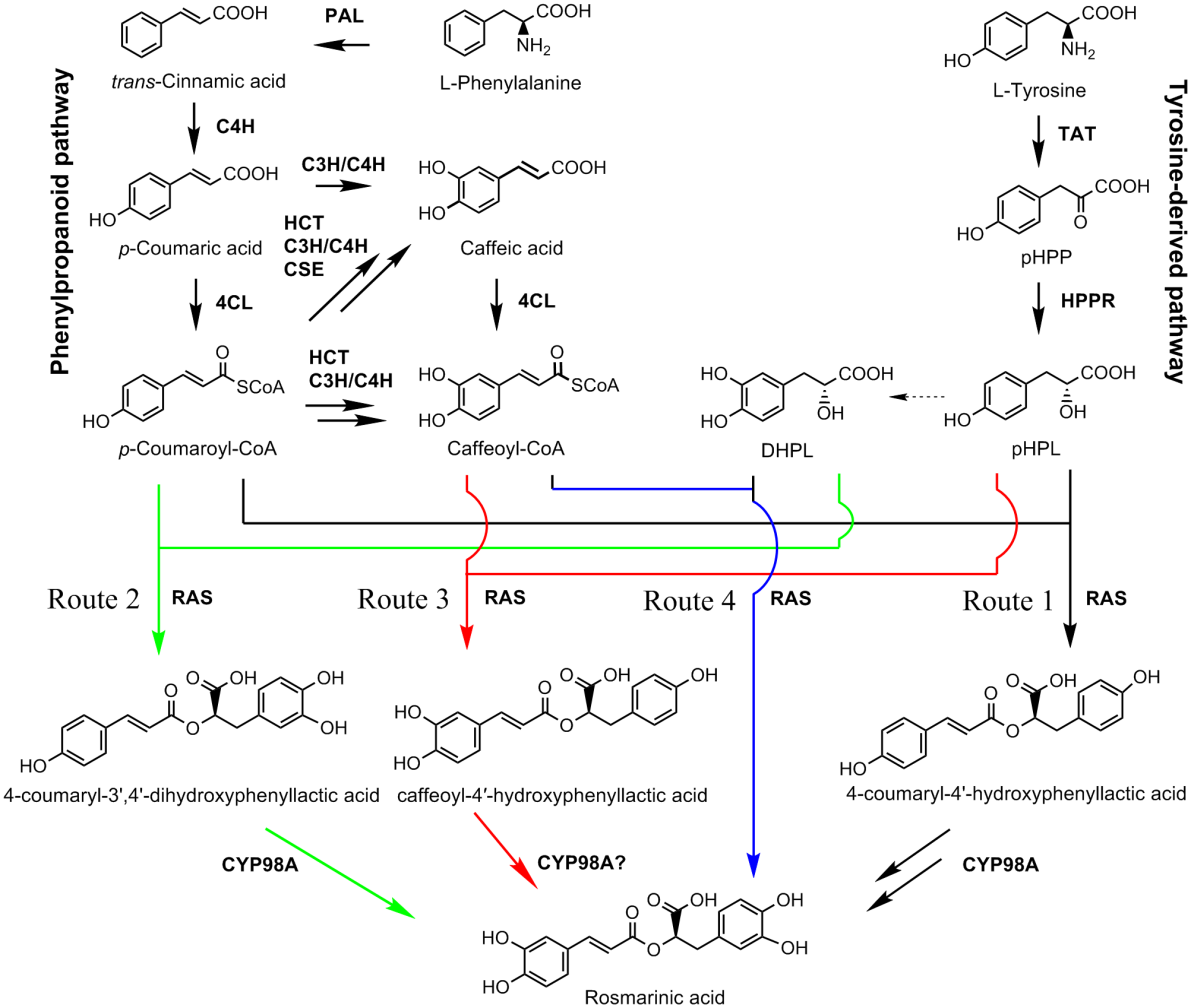
The proposed RA biosynthetic pathways (Updated from Lu, 2021). Solid arrow represents single biosynthetic step. Two arrows represent two or more steps. Dashed arrow indicates the enzyme involved in the reaction is unknown. Four proposed biosynthetic routes of RA are shown in black, green, red, and blue, respectively. Route 1 was found in *C. blumei* and *P. campanularia* (Eberle et al., 2009; Petersen et al., 2009; Levsh et al., 2019). Routes 2 and 3 were found in *S. miltiorrhiza* (Di et al., 2013; Liu et al., 2019). Route 4 was proposed in this study. 4CL, 4-coumaroyl CoA ligase; C3H, *p*-coumaroyl shikimate 3’-hydroxylase/coumarate 3-hydroxylase; C4H, cinnamate 4-hydroxylase; CSE, caffeoyl shikimate esterase; DHPL, 3,4-dihydroxyphenyllactate (danshensu); HCT, *p*-hydroxycinnamoyl-CoA: shikimate *p*-hydroxycinnamoyltransferase; HPPR, p-hydroxyphenylpyruvate reductase; PAL, phenylalanine ammonia lyase; pHPL, *p*-hydroxyphenyllactic acid; pHPP, *p*-hydroxyphenylpyruvic acid; RAS, RA synthase; TAT, tyrosine aminotransferase.

RA is a depside condensed from two single phenolic acids (Scarpati and Oriente, 1958). One of them is derived from the general phenylpropanoid pathway. It serves as the acyl donor during condensation. The other one is come from the tyrosine-derived pathway. It serves as the acyl acceptor (**Figure 1**). RA is present in some hornworts, ferns, and multiple taxa of flowering plants and its biosynthetic pathways are probably evolved independently in differently species (Petersen et al., 2009; Levsh et al., 2019). Analysis of RA biosynthesis in *Coleus blumei*, *Phacelia campanularia* and *Salvia miltiorrhiza* showed the existence of three proposed RA biosynthetic routes in different plants (**Figure 1**), which include the biosynthesis of 4-coumaroyl-4’-hydroxyphenyllactic acid from *p*-coumaroyl-CoA and *p*-hydroxyphenyllactic acid (pHPL) in *C. blumei* and *P. campanularia* (route 1), the biosynthesis of 4-coumaroyl-3’,4’-dihydroxyphenyllactic acid from *p*-coumaroyl-CoA and 3,4-dihydroxyphenyllactic acid (danshensu, DHPL) in *S. miltiorrhiza* (route 2), and the biosynthesis of caffeoyl-4’-hydroxyphenyllactic acid from caffeoyl-CoA and pHPL in *S. miltiorrhiza* (route 3) (Eberle et al., 2009; Petersen et al., 2009; Di et al., 2013; Levsh et al., 2019; Liu et al., 2019; Lu, 2021). The biosynthetic routes of RA in *P. vulgaris* are largely unknown.

Recently, we sequenced and assembled the genome of *P. vulgaris*, which provide a solid foundation for analyzing RA biosynthetic routes in *P. vulgaris* (Zhang et al., 2024). In this study, a total of 51 P*. vulgaris* genes belonging to seven RA biosynthesis-related gene families were systematically studied through genome-wide identification, feature analysis, expression analysis, and phylogenetic analysis. Among them, seventeen were identified as candidate genes for RA biosynthesis. *In vitro* enzymatic assay of PvRAS3 and PvRAS4, *in vivo* phenolic acid compound determination and *PvRAS3* transgenic analysis showed that PvRAS3 was the main enzyme catalyzing the condensation of acyl donors and acceptors during RA biosynthesis, whereas PvRAS4 played a minor role.

## Materials and methods

### Plant materials and growth conditions

A wild and whole genome sequenced *Prunella vulgaris* L. line, named Bangshan-XKC, was transplanted from Bangshan village, Shunchang county of Fujian Province of China and grown in a greenhouse at the Institute of Medicinal Plant Development in Beijing of China. Shoots were cut from the plant and surface-sterilized using 75% ethanol for 1 min and 5% sodium hypochlorite for 20 min. Subsequently, the shoots were rinsed three times with sterile water and inserted into MS medium supplemented with 30 g L^-1^ sucrose with pH value adjusted to 5.8. After two weeks, the resulting sterile plantlets were transferred to a fresh MS medium. To induce rooting, the apical and axillary buds were cut and placed on 1/2 MS medium containing 0.1 mg L^-1^ indole-3-butyric acid (IBA). The sterile plantlets were sub-cultivated in a tissue culture room on 1/2 MS medium supplemented with 30 g L^-1^ sucrose under a 16/8 h light/dark photoperiod at 25°C.

### Sequence retrieval and gene prediction

The deduced amino acid sequences of RA biosynthesis-related genes from *S. miltiorrhiza* were downloaded from NCBI GenBank (https://www.ncbi.nlm.nih.gov/protein) (Wang et al., 2015). BLAST analysis of the downloaded proteins against the chromosome-level assembly of *P. vulgaris* was carried out using the tBLASTn algorithm (Altschul et al., 1997; Zhang et al., 2024). An *E*-value cut-off of 10^-5^ was applied to the homologue recognition. Gene models were predicted from the retrieved *P. vulgaris* genomic DNA sequences based on the downloaded *S. miltiorrhiza* genes and through BLASTx analysis of retrieved sequences against the NR database using the default parameters (https://blast.ncbi.nlm.nih.gov/Blast.cgi). The predicted gene models were further examined and corrected manually through BLASTn analysis against *P. vulgaris* transcriptome sequencing data (Altschul et al., 1997; Zhang et al., 2024).

### Gene and protein feature analysis

The theoretical isoelectric point (p*I*) and molecular weight (Mw) were calculated using the Compute pI/MW tool on the ExPASy server (https://web.expasy.org/compute_pi/). Protein subcellular localization was predicted using Plant-mPLoc version 2.0 (http://www.csbio.sjtu.edu.cn/bioinf/plant-multi/#). The number of transmembrane regions was predicted using DeepTMHMM version 1.0.24 (https://dtu.biolib.com/DeepTMHMM). Distribution of genes on the chromosomes of *P. vulgaris* was visualized using TBtools (Chen et al., 2020). Intron/exon structures were predicted using GSDS2.0 on the Gene Structure Display Server (http://gsds.gao-lab.org/).

### Phylogenetic analysis

RA biosynthesis-related protein sequences from various plant species were downloaded from NCBI GenBank (https://www.ncbi.nlm.nih.gov/protein). Sequence alignment was carried out using the ClustalW algorithms in MEGA version 7.0.26 (Kumar et al., 2016). Neighbor-joining trees were constructed for amino acid sequences using MEGA versopm 7.0.26 with default parameters (Kumar et al., 2016). The number of bootstrap replications was 1000.

### Quantitative real-time PCR analysis of gene expression

Total RNA was extracted from roots, stems, leaves and spikes using the EASYspin Plus Complex Plant RNA kit (Aidlab, China) as described previously (Cui et al., 2022). Genomic DNA contamination was eliminated by treating with RNase-free DNase (Aidlab, China). RNA integrity was evaluated on a 1% argarose gel. RNA quantity was determined using a NanoDrop 2000C Spectrophotometer (Thermo Scientific, USA). RNA was reverse-transcribed into single-stranded cDNA using Superscript III Reverse Transcriptase (Invitrogen, USA). qRT-PCR was carried out using TB Green Premix Ex Taq II (Takara, Japan) on a Bio-Rad CFX96 Real-Time system. Primers used for qRT-PCR were designed using Primer Premier 5 (Lalitha, 2000) and are shown in **Supplementary Table 1**. Gene amplification efficiency of each primer pair was evaluated using the standard curves. Primer pairs with an appropriate PCR amplification efficiency were used for subsequent analysis (**Supplementary Figure 1**). *PveIF-2* was selected as the reference gene as described before (Zheng et al., 2022). The specificity of amplification was assessed by dissociation curve analysis. Relative abundance of transcripts was determined using the 2^-ΔΔCt^ method. Standard deviations were calculated from three biological replicates and three PCR replicates per biological replicates.

### Analysis of gene expression using RNA-seq data

Gene expression was analyzed using the published transcriptome data from roots, stems, leaves, and spikes (Zhang et al., 2024). Salmon software (v1.10.3) (Sahraeian et al., 2017) was used to quantify the level of gene expression. Heat maps were constructed using the TBtools software (Chen et al., 2020).

### *PvRAS3* and *PvRAS4* gene cloning and expression vector construction

Total RNA extracted from leaves of *P. vulgaris* was reverse-transcribed into cDNA using the SuperScript III First-Strand Synthesis System for RT-PCR (Invitrogen, USA). *PvRAS3* and *PvRAS4* were amplified by nested PCR using cDNA from *P. vulgaris* leaves as the template. The nesting and nested primers used for PCR are listed in **Supplementary Table 1**. PCR products were inserted into pGEX-4T-1 and verified by Sanger sequencing.

### Heterologous expression of PvRAS3 and PvRAS4 proteins in *E. coli*

The pGEX-4T-1 vector with *PvRAS3* or *PvRAS4* was introduced into *E. coli* strain BL21 (DE3). Heterologous expression of PvRAS3 and PvRAS4 proteins were induced with 0.5 mmol L^-1^ IPTG at 16 °C for 20–24 h. Cells were collected through centrifugation at 6,000 rpm for 10 min at 4 °C. After resuspension in 10 mM PBS buffer (pH 7.2), the cells were sonicated on ice. Purification of soluble proteins was carried out using the PurKine™ GST-Tag Protein Purification kit (Glutathione) (Abbkine, China). Concentration of the purified proteins was determined using the BCA Protein Assay kit (Takara Biomedical Technology, Beijing).

### *In vitro* enzymatic activity assay of PvRAS3 and PvRAS4 recombinant proteins

The enzymatic activity assay was carried out in a 500 µl reaction system comprising 100 µg purified proteins, 1 mM caffeoyl-CoA or *p*-coumaroyl-CoA as the acyl donors, 1 mM pHPL or DHPL as the acyl receptors. The reactions were incubated at 25 °C for 60 min and terminated by adding 10 µl of 10 M acetic acid. Controls were carried out using total proteins from *E. coli* transformed with the empty pGEX-4T-1 vector. Reaction products were collected and analyzed using ACQUITY UPLC system (Waters, Milford, MA, USA). MS/MS data were recorded on a Xevo G2-XS Q-ToF Mass Spectrometer (Waters, Milford, MA, USA) coupled to a Waters Acquity I-Class UPLC system (Waters, Milford, MA, USA). MS/MS analyses were conducted in negative-ion mode. The samples were separated on an ACQUITY UPLC BEH C18 column (1.7 *μ*m, 100×2.1 mm) at 25°C. The mobile phase A was 0.1% (v/v) formic acid-acetonitrile. The mobile phase B was 0.1% (v/v) formic acid in water. The flow rate was 0.3 mL min^−1^. The mobile phases changed with the following gradient: 0–6 min, 5% A and 95% B; 6–8 min, 20% A and 80% B; 8–14 min, 21% A and 79% B; 14–18 min, 95% A and 5% B. MS was analyzed using electrospray ionization (ESI) at negative ion mode. MS-MS data were analyzed using the MssLynx V4.1 software (Waters) as described previously (Pan et al., 2023).

### Kinetic analysis of PvRAS3 and PvRAS4 recombinant proteins

Kinetic analysis of PvRAS3 and PvRAS4 was carried out in a 200 μL reaction system consisting of Tris-HCl buffer (100 mM Tris-HCl, pH 7.0, 2 mM DTT, 4 mM MgCl_2_, 10% glycerol), 100 μg recombinant protein, and different concentrations of substrates. The reactions were incubated at 25 °C for 30 min and terminated by adding 10 *µ*l of 10 M acetic acid. The reaction products were analyzed using UPLC system as described as *in vitro* enzymatic activity assay of recombinant proteins. Enzyme activity was determined by measuring the variation of substrate contents. To determine kinetic parameters, PvRAS3 or PvRAS4 was incubated with different concentrations of acyl donor and acyl acceptor. The saturation concentration of one substrate was set at 2 mM, while the concentration of another substrate was varied at different levels, including 10 μM, 50 μM, 100 μM, 250 μM, 350 μM, 500 μM, and 1000 μM, respectively. The kinetic constants of the donor substrates were calculated based on contents of the product. The kinetic constants of the acceptor substrates were determined through monitoring the consumption of the acceptor substrates. Enzyme assays were performed in triplicate at each concentration of substrate. *V*max and *K*m values were calculated using Origin 8.0 software with nonlinear regression analysis.

### UPLC and LC-MS/MS analyses of phenolic acids

Roots, stems, leaves and spikes of two-year-old *P. vulgaris* were ground in liquid nitrogen. The ground samples (0.5 g) were dissolved in 10 ml of 80% ethanol and sonicated for 60 min. The extracts were collected by centrifugation and filtered using a 0.22 μm filter (Merk Millipore, USA). UPLC and LC-MS/MS analyses were performed using the ACQUITY UPLC I-Class system (Waters) as described as *in vitro* enzymatic activity assay of recombinant proteins. Three biological and three technological replicates were carried out for analysis of each tissue.

### Generation and analysis of *pvras3* mutants

*Pvras3* mutants of *P. vulgaris* hairy roots were generated using the CRISPR/Cas9 system described previously (Wang et al., 2022a). Briefly, PCR amplification was carried out using two pairs of primers containing two dividual guide RNAs (sgRNAs) sequences of *PvRAS3*. pDT1T2 vector was used as a template. The products were purified, digested with *Bsa* I, and ligated into the binary vector pHEE401E. The resulting constructs were transferred into *Agrobacterium* strain ATCC15834.

Leaf discs from thirty-day-old sterile plantlets were cultivated on 1/2 MS medium in dark for two days, immersed for 10 min in the suspension of *Agrobacterium* cells with or without the constructs, and co-cultivated on MS medium for 2 days. The leaf discs were then transferred onto 1/2 MS medium supplemented with 30 mg L^-1^ of hygromycin and 400 mg L^-1^ of cefotaxime for generation of hairy roots. Leaf discs were subcultured every two weeks. Hairy roots generated were transferred to 1/2 MS medium supplemented with 200 mg L^-1^ of cefotaxime and cultivated for about two weeks. Newly generated hairy roots were then transferred to 1/2 MS medium supplemented with 100 mg L^-1^ of cefotaxime and cultivated for about two weeks. Finally, newly generated hairy roots from medium with 100 mg L^-1^ of cefotaxime were transferred to 1/2 MS medium without cefotaxime and cultivated for two weeks. Root tips with 3-4 cm in length were cut, transferred to 100 ml of 1/2 MS medium in 250 ml-flasks, and cultivated at 25°C in dark with 100 rpm shaking.

To analyze the mutations of *PvRAS3* in transgenic hairy roots, genomic DNA was extracted. DNA fragments around the target site were PCR-amplified using gene-specific primers, Mut-F: GTCGTTTGCTCCCTTACAAAT, and Mut-R: GATCGAAGTGAAGGAGTCGACG. PCR products were sequenced using the primer Mut-F. Hairy roots generated from leaf discs through inoculation with *Agrobactrium* without the constructs were used as a control. UPLC analysis of chemical compounds was performed using the ACQUITY UPLC I-Class system (Waters). Three biological and three technological replicates were carried out for analysis of each transgenic hairy root line.

## Results and discussion

### Genome-wide identification of genes associated with RA biosynthesis in *P. vulgaris*

RA is synthesized through the general phenylpropanoid pathway and the tyrosine-derived pathway, involving at least nine enzymes encoded by seven gene families (**Figure 1**) (Deng and Lu, 2017; Lu, 2021). In order to identify *P. vulgaris* genes involved in RA biosynthesis, tblastn analysis of the deduced protein sequences of RA biosynthesis-related genes in *Salvia miltiorrhiza* against the whole-genome assembly of *P. vulgaris* (2n=28) was carried out (Altschul et al., 1997; Wang et al., 2015; Zhang et al., 2024). It resulted in the identification of 51 full-length candidate genes, including four putative *PvPALs*, three putative *PvC4Hs*, seventeen putative *Pv4CLs*, seven putative *PvTATs*, four putative *PvHPPRs*, three putative *PvHCTs*, eight putative *PvRASs*, and five putative *PvCYP98As* (**Table 1**). These genes were designated as *PvPAL1*–*PvPAL4*, *PvC4H1*–*PvC4H3*, *Pv4CL1*–*Pv4CL17*, *PvTAT1*–*PvTAT7*, *PvHPPR1*–*PvHPPR4*, *PvHCT1*–*PvHCT3*, *PvRAS1*–*PvRAS8*, and *PvCYP98A-1*–*PvCYP98A-5*, respectively. Among them, *PvPAL1* and *PvC4H1* showed 99% identities at the amino acid level with the reported *PvPAL* (KJ010815) and *PvC4H* (KJ010816), respectively (Kim et al., 2014). *Pv4CL3* showed 97% identities at the amino acid level with the reported *Pv4CL1* (KJ010814), whereas *Pv4CL8* showed 99% amino acid identities with the reported 5’ truncated *Pv4CL2* (KJ010817) (Kim et al., 2014). *PvTAT3*, PvRAS3 and PvCYP98A-1 showed 99% identities at the amino acid level with the reported *PvTAT* (M053278), *PvRAS* (KM053280) and *PvCYP98A101* (AJW87635), respectively (Ru et al., 2017a; Ru et al., 2017b). *PvHPPR3* was identical to the sequence under the GenBank accession number KM053279. The other 43 genes have not been reported previously.

**Table 1:** Sequence features of RA biosynthesis-related genes in *P. vulgaris*.

Gene name	Gene Length (bp)	ORF length (bp)^1^	Protein length (aa)	p*I* ^2^	Mw (kDa)^3^	Loc^4^	TMR^5^
*PvPAL1*	2,699	2,130	710	6.06	76.62	Cyt	0
*PvPAL2*	2,676	2,127	709	5.84	77.01	Cyt	0
*PvPAL3*	3,224	2,121	707	6.04	76.87	Cyt	0
*PvPAL4*	2,732	2,127	709	5.9	76.83	Cyt	0
*PvC4H1*	3,081	1,515	505	9.22	57.94	ER	0
*PvC4H2*	3,062	1,515	505	9.17	57.84	ER	0
*PvC4H3*	2,200	1,509	503	9.21	57.02	ER	0
*Pv4CL1*	2,866	1,620	540	5.5	58.62	Per	0
*Pv4CL2*	3,719	1,620	540	6.62	58.44	Per	0
*Pv4CL3*	3,115	1,695	565	5.48	60.78	Per	0
*Pv4CL4*	4,608	1,671	557	5.95	60.04	Per	0
*Pv4CL5*	4,069	1,680	560	6.33	60.3	Per	0
*Pv4CL6*	3,650	1,695	565	5.42	60.7	Per	0
*Pv4CL7*	4,143	1,647	549	5.95	59.73	Per	0
*Pv4CL8*	3,820	1,632	544	5.84	59.02	Per	0
*Pv4CL9*	1,962	1,695	565	5.87	61.55	Per	0
*Pv4CL10*	3,338	1,635	545	8.75	59.46	Per	0
*Pv4CL11*	3,773	1,662	554	8.39	59.99	Per	0
*Pv4CL12*	2,488	1,722	574	7.28	62.33	Per	0
*Pv4CL13*	4,674	1,605	535	8.59	58.67	Per	0
*Pv4CL14*	1,930	1,692	564	5.82	61.8	Per	0
*Pv4CL15*	2,806	1,722	574	6.77	62.51	Per	0
*Pv4CL16*	3,290	1,620	540	7.24	59.16	Per	0
*Pv4CL17*	3,430	1,608	536	6.97	58.73	Per	0
*PvTAT1*	1,759	1,206	402	7.06	44.33	Chl	0
*PvTAT2*	2,707	1,263	421	5.88	45.95	Chl	0
*PvTAT3*	2,627	1,233	411	5.8	45.11	Chl	0
*PvTAT4*	2,421	1,233	411	5.8	45.05	Chl	0
*PvTAT5*	1,997	1,308	435	6.23	48.49	Chl	0
*PvTAT6*	1,904	1,251	417	7.61	46	Chl	0
*PvTAT7*	2,498	1,281	427	5.77	47.18	Chl	0
*PvHPPR1*	1,818	927	309	5.29	34.04	M	0
*PvHPPR2*	2,026	939	313	5.99	33.99	M	0
*PvHPPR3*	1,716	939	313	5.66	34	M	0
*PvHPPR4*	1,459	957	319	7.74	35.09	M	0
*PvHCT1*	1,640	1,281	427	5.63	47.12	Cyt	0
*PvHCT2*	3,296	1,281	427	5.73	47.33	Cyt	0
*PvHCT3*	6,909	1,281	427	6.47	47.28	Cyt	0
*PvRAS1*	1,528	1,299	433	6.51	48.14	Cyt	0
*PvRAS2*	1,411	1,326	442	6.64	49.22	Cyt	0
*PvRAS3*	2,635	1,305	435	5.97	48.17	Cyt	0
*PvRAS4*	1,281	1,278	426	6.14	47.41	Cyt	0
*PvRAS5*	1,599	1,305	435	6.94	48.37	Cyt	0
*PvRAS6*	3,031	1,293	431	6.47	48.08	Cyt	0
*PvRAS7*	4,937	1,293	431	6.15	48.13	Cyt	0
*PvRAS8*	3,331	1,308	436	6.08	48.71	Cyt	0
*PvCYP98A-1*	2,357	1,530	510	8.62	57.76	ER	0
*PvCYP98A-2*	2,304	1,533	511	9.12	58.23	ER	0
*PvCYP98A-3*	1,811	1,533	511	8.61	57.86	ER	0
*PvCYP98A-4*	2,490	1,530	510	8.13	57.68	ER	0
*PvCYP98A-5*	2,703	1,530	510	8.13	57.84	ER	0

^1^ORF stands for the open reading frame of a gene;

^2,3^p*I* and molecular weight (Mw) were calculated using the Compute pI/MW tool on the ExPASy server (https://web.expasy.org/compute_pi/);

^4^Loc represents protein subcellular localization predicted using Plant-mPLoc version 2.0 (http://www.csbio.sjtu.edu.cn/bioinf/plant-multi/#).’ Cyt’, ‘ER’, ‘Per’, ‘Chl’ and ‘M’stand for cytoplasm, endoplasmic reticulum, peroxisome, chloroplast and mitochondrion, respectively.

^5^TMR represents the number of transmembrane regions predicted using DeepTMHMM version 1.0.24 (https://dtu.biolib.com/DeepTMHMM).

The identified genes are distributed on the 14 chromosomes of the whole genome assembly of *P. vulgaris* (**Supplementary Figure 2**) (Zhang et al., 2024). The deduced proteins have length varying from 309 to 709 amino acid residues, p*I* varying from 5.29 to 9.22, and molecular weight varying from 34.00 to 77.01 (**Table 1**). All of them do not contain transmembrane regions and were predicted to be localized in the cytoplasm, endoplasmic reticulum, peroxisome, chloroplast and mitochondrion, respectively (**Table 1**). The predicted localization of PvPALs, PvHCTs and PvRASs in the cytoplasm is consistent with the experimental results from tobacco, *S. miltiorrhiza* (Achnine et al., 2004; Di et al., 2013). The predicted localization of PvC4Hs and PvCTP98As in the endoplasmic reticulum is consistent with the experimental results from *Populus*, *S. miltiorrhiza*, and *P. vulgaris* (Ro et al., 2001; Di et al., 2013; Ru et al., 2017b). Pv4CLs, PvTATs and PvHPPRs were predicted to be localized in the peroxisome, chloroplast and mitochondrion, respectively. However, *Peucedanum praeruptorum* 4CL, *P. vulgaris* TAT and *S. miltiorrhiza* HPPR were previously found to be located in the cytoplasm (Liu et al., 2017; Wang et al., 2017; Ru et al., 2017a). Thus, the actual subcellular localization of Pv4CLs, PvTATs and PvHPPRs remain to be experimentally analyzed.

### Characterization and expression analysis of genes involved in the general phenylpropanoid pathway

**Figure 2:**
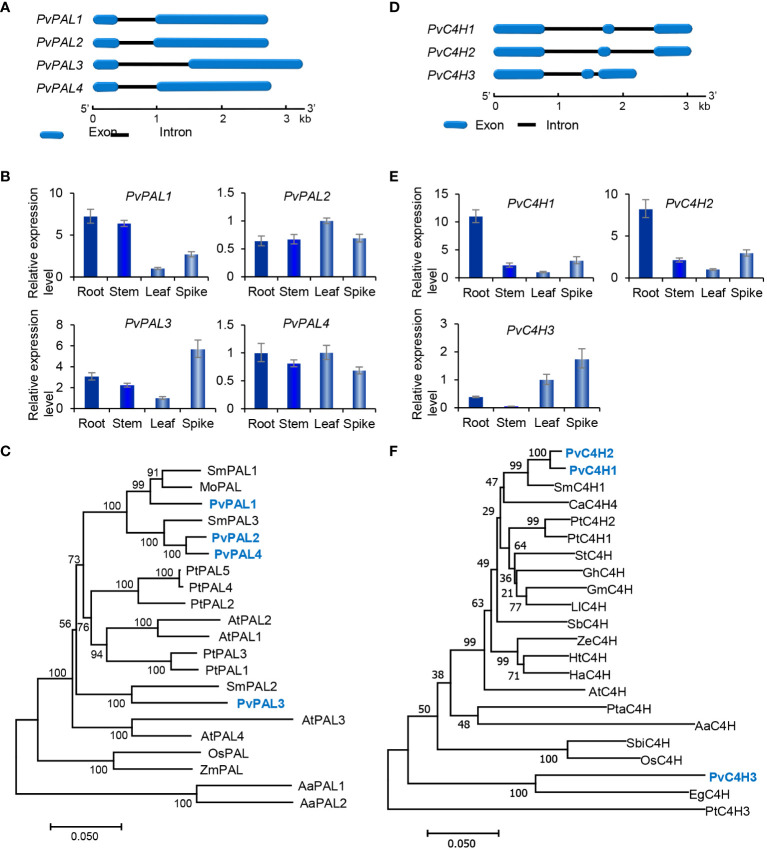
Gene structures, expression patterns and phylogenetic analysis of *PvPAL* and *PvC4H* genes and their deduced proteins. **(A, D)** The intron-exon structures of *PvPAL* **(A)** and *PvC4H* **(D)** genes. **(B, E)** Fold changes of *PvPAL* **(B)** and *PvC4H* **(E)** gene expression in roots, stems, leaves and spikes of *P. vulgaris* plants. The expression level in leaves was arbitrarily set to 1, respectively. **(C)** Phylogenetic analysis of PAL proteins. The rooted Neighbor-Joining tree was constructed using the MEGA program (version 7.0) with default parameters. AaPAL1 (QPI70499.1) and AaPAL2 (SPO49995.1) from *Anthoceros agrestis* were used as outgroup. Ingroup consists of four PvPALs and the PALs from *S. miltiorrhiza* (Sm), *Arabidopsis* (At), *Populus trichocarpa* (Pt), *Melissa officinalis* (Mo), and maize (Zm) (**Supplementary Table 2**). **(F)** Phylogenetic analysis of C4H proteins. C4Hs included are three PvC4Hs and the C4Hs from *S. miltiorrhiza* (Sm), *Coffea Arabica* (Ca), *P. trichocarpa* (Pt), *Solanum tuberosum* (St), *Gossypium hirsutum* (Gh), *Glycine max* (Gm), *Leucaena leucocephala* (Ll), *Scutellaria baicalensis* (Sb), *Zinnia elegans* (Ze), *Helianthus tuberosus* (Ht), *Helichrysum aureonitens* (Ha), *Arabidopsis* (At), *Pinus taeda* (Pt), *A*. *agrestis* (Aa), *Sorghum bicolor* (Sb), rice (Os), and *Erythranthe guttata* (Eg) (**Supplementary Table 2**).

**Figure 3:**
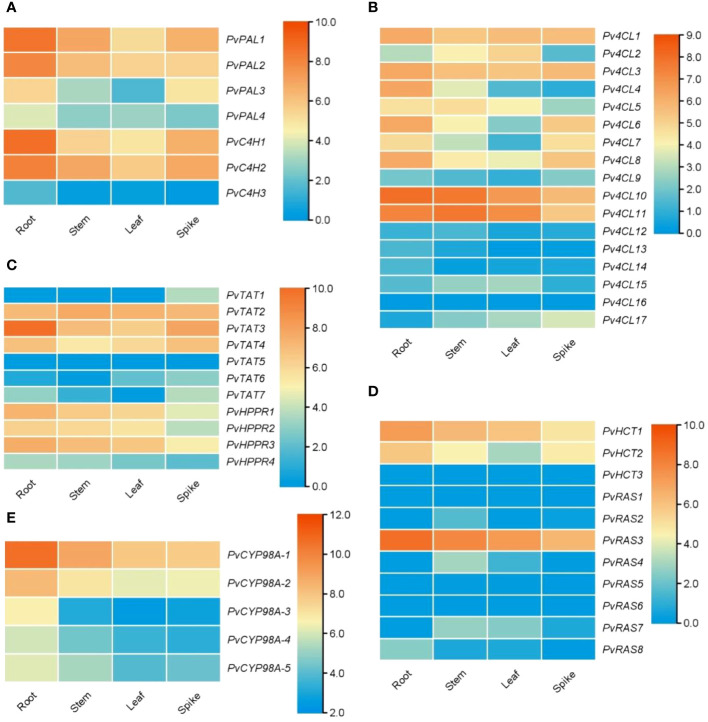
Expression of RA biosynthesis-related genes in roots, stems, leaves and spikes of *P. vulgaris*. **(A–E)** Hierarchical clustering of the expression levels of RA biosynthesis-related genes analyzed using RNA-seq clean data.

The general phenylpropanoid pathway involves three enzymes, including phenylalanine ammonia lyase (PAL), cinnamate 4-hydroxylase (C4H), and 4-coumaroyl CoA ligase (4CL) (**Figure 1**). PAL catalyzes the conversion of L-phenylalanine to *trans*-cinnamic acid through deamination of L-phenylalamine. It is the first reaction of the general phenylpropanoid pathway and a rate limiting step mediating the influx from primary metabolism into the general phenylpropanoid pathway (Raes et al., 2003). In a plant, PAL is usually encoded by a small gene family. For instance, there are two *PAL* genes in tobacco, three in *S. miltiorrhiza*, and four in *Arabidopsis* (Raes et al., 2003; Achnine et al., 2004; Wang et al., 2015). Genome-wide analysis showed that there were four putative *PvPAL* genes in *P. vulgaris* (**Table 1**), all of which contained an intron and had similar gene structures (**Figure 2A**). qRT-PCR analysis showed that the four *PvPAL* genes were differentially expressed (**Figure 2B**). RNA-seq analysis showed that *PvPAL1* and *PvPAL2* were expressed relatively higher than the other two *PvPALs* (**Figure 3A**). The expression patterns revealed between qRT-PCR and RNA-seq were largely consistent for *PvPALs* and other genes analyzed hereinafter. However, discrepancy was also observed, which could be results from the difference of detection technologies, plant tissues, data analysis method, or other unknown factors. Analysis of the deduced PvPAL proteins showed that all of them contained the conserved “GTITASGDLVPLSYIA” motif involved in substrate binding and catalytic activity and the conserved “FL” residues impartent for substrate specificity (Poppe and Rétey, 2005; Watts et al., 2006; Ma et al., 2013). In addition, other three conserved catalytic active sites, including “GLALVNG”, “NDN” and “HNQD”, were also found (**Supplementary Figure 3**) (He et al., 2020). It indicates that all of the four identified PvPALs have catalytic activity. Phylogenetic analysis of PALs from *P. vulgaris*, *S. miltiorrhiza*, *Arabidospsis*, *Populus trichocarpa* and various other plant showed that PvPAL1, PvPAL2 and PvPAL4 were grouped with SmPAL1, SmPAL3 and MoPAL involved in RA biosynthesis (**Figure 2C**) (Weitzel and Petersen, 2010; Song and Wang, 2011; Hou et al., 2013). Taken together with previous results for PvPAL1 (Kim et al., 2014), the presence of RA in roots, stems, leaves and flowers (Kim et al., 2014), and the results from gene expression analysis, we speculated that PvPAL1 and PvPAL2 could be the main PvPALs for RA biosynthesis.

C4H catalyzes the hydroxylation of *trans*-cinnamic acid to *p*-coumaric acid (**Figure 1**). It is encoded by the members of CYP73A gene subfamily. Genome-wide analysis showed that there were three putative *PvC4H* genes in *P. pulgaris*. All of them contained two introns (**Figure 2D**). *PvC4H1* and *PvC4H2* showed similar expression patterns with the highest expression in roots, followed by spikes, stems and leaves (**Figures 2E**, **3A**). High expression of *PvC4H1* and *PvC4H2* is consistent with high content of RA in roots, stems, leaves and flowers (Kim et al., 2014). The expression of *PvC4H3* was very low in four tissues analyzed (**Figure 3A**). Analysis of the deduced proteins showed that all of the three PvC4Hs contained five conserved P450 motifs, including the proline-rich motif “PPGP”, the oxygen binding motif “AAIETT”, the “ETLR” motif, the “PERF” motif, and the heme-binding motif “FGVGRRSCPG” (**Supplementary Figure 4**) (Khatri et al., 2023). Phylogenetic analysis of C4Hs from *P. vulgaris*, *S. miltiorrhiza*, *Arabidospsis*, *P. trichocarpa* and various other plants showed that PvC4H1 and PvC4H2 were grouped with SmC4H1 involved in RA biosynthesis (**Figure 2F**), indicating the involvement of PvC4H1 and PvC4H2 in RA biosynthesis (Xiao et al., 2011; Kim et al., 2014; Wang et al., 2015).

**Figure 4:**
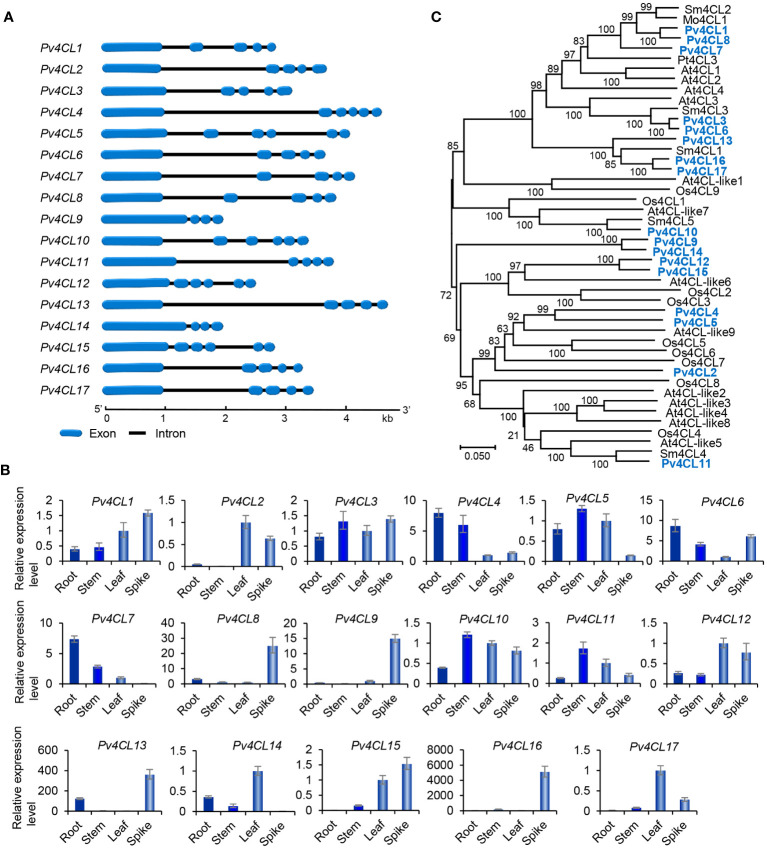
Gene structures, expression patterns and phylogenetic analysis of *Pv4CL* genes and their deduced proteins. **(A)** The intron-exon structures of *Pv4CL* genes. **(B)** Fold changes of *Pv4CL* gene expression in roots, stems, leaves and spikes of *P. vulgaris* plants. The expression level in leaves was arbitrarily set to 1, respectively. **(C)** Phylogenetic analysis of 4CL proteins. The unrooted Neighbor-Joining tree was constructed using the MEGA program (version 7.0) with default parameters. 4CLs included are seventeen Pv4CLs and other 4CLs from *S. miltiorrhiza* (Sm), *Arabidopsis* (At), rice (Os), *M. officinalis* (Mo), and *P. trichocarpa* (Pt) (**Supplementary Table 2**).

4CL is the third and the last enzyme of the general phenylpropanoid pathway. It catalyzes the thioesterification of *p*-coumaric acid (**Figure 1**). The product, *p*-coumaroyl-CoA, can be funneled into downstream branch pathways for lingnins, flavonoids, coumarins, lignans, and RA (Deng and Lu, 2017). 4CL is encoded by a multiple gene family. For instance, there are seventeen *Pt4CLs* in *P. trichocarpa*, ten *Sm4CLs* in *S. miltiorrhiza*, and thirteen *At4CLs* and *At4CL-likes* in *Arabidopsis* (Raes et al., 2003; Shi et al., 2010; Wang et al., 2015). Genome-wide analysis showed that there were seventeen putative *Pv4CL* genes with 3–5 introns in *P. pulgaris* (**Figure 4A**). Gene expression analysis showed that the seventeen *Pv4CLs* had differential expression patterns (**Figure 4B**). RNA-seq analysis showed that the levels of *Pv4CL1*, *Pv4CL3*, *Pv4CL8*, *Pv4CL10* and *Pv4CL11* were relatively high, whereas the levels of *Pv4CL9* and *Pv4CL12*–*Pv4CL17* were very low in the four tissues analyzed (**Figure 3B**).

It is generally known that the 4CL proteins contain two conserved motifs, including Box I with the representative sequence “SSGTTGLPKGV” and Box II with the representative sequence “GEICIRG” (Uhlmann and Ebel, 1993). Box I is conserved in adenylate-forming enzymes and involved in adenosine monophosphate (AMP)-binding. Box II is conserved in 4CL and related to the spatial conformation of the enzyme (Wang et al., 2022b). Sequence alignment of the seventeen Pv4CL proteins showed that Pv4CLs also had the two conserved motifs. However, their sequences were divergent (**Supplementary Figure 5**). It indicates that the identified seventeen Pv4CLs could be functionally diverse. Phylogenetic analysis of 4CLs from *P. vulgaris*, *S. miltiorrhiza*, *Arabidospsis*, rice and various other plants showed that Pv4CL1, Pv4CL3, Pv4CL6, Pv4CL7 and Pv4CL8 were grouped with Mo4CL1, Sm4CL2 and Sm4CL3 involved in RA biosynthesis (**Figure 4C**) (Zhao et al., 2006; Weitzel and Petersen, 2010; Wang et al., 2015). These Pv4CLs could be associated with RA biosynthesis in *P. vulgaris* (Kim et al., 2014).

### Characterization and expression analysis of genes involved in the tyrosine-derived pathway

**Figure 5:**
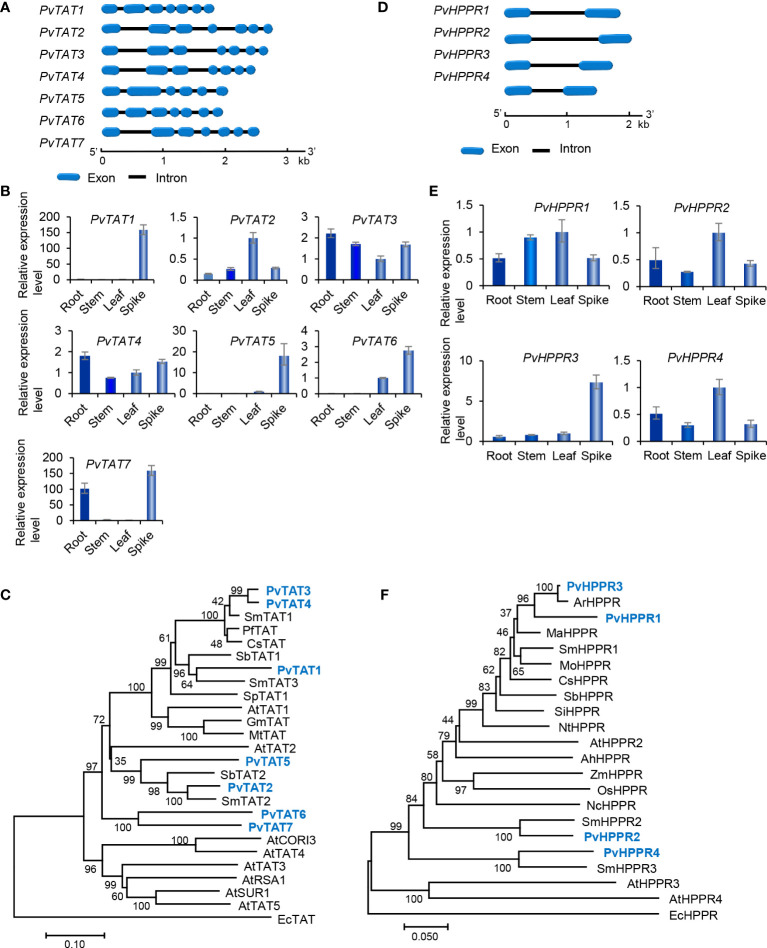
Gene structures, expression patterns and phylogenetic analysis of *PvTAT* and *PvHPPR* genes and their deduced proteins. **(A, D)** The intron-exon structures of *PvTAT* **(A)** and *PvHPPR* **(D)** genes. **(B, E)** Fold changes of *PvTAT* **(B)** and *PvHPPR* **(E)** gene expression in roots, stems, leaves and spikes of *P. vulgaris* plants. The expression level in leaves was arbitrarily set to 1, respectively. **(C)** Phylogenetic analysis of TAT proteins. The rooted Neighbor-Joining tree was constructed using the MEGA program (version 7.0) with default parameters. EcTAT (NP_418478.1) from *Escherichia coli* was used as outgroup. Ingroup TATs include PvTAT1–PvTAT7 and others from *S. miltiorrhiza* (Sm), *Arabidopsis* (At), *Perilla frutescens* (Pf), *Coleus scutellarioides* (Cs), *S. baicalensis* (Sb), *Solanum pennellii* (Sp), *G. max* (Gm), and *Medicago truncatula* (Mt) (**Supplementary Table 2**). **(F)** Phylogenetic analysis of HPPR proteins. The rooted Neighbor-Joining tree was constructed using the MEGA program (version 7.0) with default parameters. EcHPPR (WP_000811015.1) from *Escherichia coli* was used as outgroup. Ingroup HPPRs include PvHPPR1–PvHPPR4 and others from *S. miltiorrhiza* (Sm), *Arabidopsis* (At), *Agastache rugosa* (Ar), *Mentha aquatica* (Ma), *M. officinalis* (Mo), *C*. *scutellarioides* (Cs), *S. baicalensis* (Sb), *Sesamum indicum* (Si), *Nicotiana tomentosiformis* (Nt), *Arachis hypogaea* (Ah), *Zea mays* (Zm), *Oryza sativa* (Os), and *Nymphaea colorata* (Nc) (**Supplementary Table 2**).

The tyrosine-derived pathway involves two enzymes, including tyrosine aminotransferase (TAT) and *p*-hydroxyphenylpyruvate reductase (HPPR) (**Figure 1**). TAT catalyzes the conversion of L-tyrosine to pHPP, a precursor for the biosynthesis of various secondary metabolites, such as plastoquinone, tocopheros, benzylisoquinoline alkaloids, and RA. In plants, TAT is encoded by multiple gene family with three members in *S. miltiorrhiza*, four in apple, and eight in *Arabidopsis* (Wang et al., 2015, 2018). Through genone-wide mining, we identified seven putative *PvTAT* genes that had five or six introns (**Figure 5A**). Among them, *PvTAT2*–*PvTAT3* showed relatively high expression (**Figures 3C**, **5B**). The expression of other four *PvTATs*, including *PvTAT1* and *PvTAT5*–*PvTAT7*, was mainly in spikes and the level was very low in the tissues analyzed, in comparison with *PvTAT2*–*PvTAT3* (**Figures 3C**, **5B**). Sequence alignment of the seven PvTAT proteins showed that all of them contained the conserved Motif 1 for aminotransferase family-I pyridoxal phosphate binding site and Motif 2 with the highly conserved residue Arg (**Supplementary Figure 6**) (Lu et al., 2013; Wang et al., 2018). Phylogenetic analysis of seven PvTATs and TATs from *S. miltiorrhiza*, *Arabidopsis* and other plants showed that PvTAT3 and PvTAT4 were clustered with SmTAT1 and PfTAT involved in RA biosynthesis (**Figure 5C**) (Xiao et al., 2011; Lu et al., 2013). PvTAT3 was previously shown to participate in the biosynthesis of RA in *P. vulgaris* and its high expression in four tissues analyzed is consistent with the accumulation of RA (**Figure 5B**) (Kim et al., 2014; Ru et al., 2017a). PvTAT4 could be a novel PvTAT playing a redundant role with PvTAT3 in RA biosynthesis.

HPPR, belonging to the family of D-isomer-specific 2-hydroxyacid dehydrogenases, is the other enzyme involved in the tyrosine-derived pathway. It catalyzes the conversion of pHPP to pHPL (Kim et al., 2004) (**Figure 1**). HPPR is encoded by a small gene family, such ast there are three *SmHPPR* genes in *S. miltiorrhiza* and four in *Arabidopsis* (Wang et al., 2015; Xu et al., 2018). Its involvement in RA biosynthesis has been verified through functional analysis of *MoHPPR* from *Melissa offcianalis* (Mansouri and Mohammadi, 2021), *CsHPPR* from *Coleus scutellarioides* (Kim et al., 2004), and *SmHPPR1* from *S. miltiorrhiza* (Xiao et al., 2011; Wang et al., 2017). Genome-wide analysis showed that there were four *PvHPPR* genes in *P. vulgaris*, all of which contained one intron and had similar structures (**Figure 5D**). All of them showed differential expression patterns, and the overall expression level of *PvHPPR1*–*PvHPPR3* was higher than *PvHPPR4* (**Figures 3C**,**5E**). Amino acid sequence alignment showed that all four PvHPPR proteins contained the NAD(P)H binding motif with the representative sequence “GLGRIG” and the putative myristylation site with the representative sequences “GTVETR” and “GNLEA” (**Supplementary Figure 7**) (Wang et al., 2017). Phylogenetic analysis of four PvHPPRs and HPPRs from *S. miltiorrhiza*, *Arabidopsis* and other plants showed that PvHPPR1 and PvHPPR3 were grouped with SmHPPR1, MoHPPR and CsHPPR involved in RA biosynthesis (**Figure 5F**). It suggests that PvHPPR1 and PvHPPR3 could be involved in the biosynthesis of RA in *P. vulgaris*. The function of PvHPPR2 and PvHPPR4 remain to be elucidated.

### Characterization and expression analysis of genes involved in downstream of the RA biosynthetic pathway

The downstream of RA biosynthetic pathway involves four known enzymes, including *p*-hydroxycinnamoyl-CoA: shikimate *p*-hydroxycinnamoyltransferase (HCT), rosmarinic acid synthase (RAS), *p*-coumaroyl shikimate 3’-hydroxylase/coumarate 3-hydroxylase (C3H), and the enzyme catalyzing the final step of RA biosynthetic pathway (**Figure 1**). Enzymes involved in DHPL biosynthesis are currently unknown (**Figure 1**). Among the four known enzymes, HCT catalyzes the coupling of *p*-coumaroyl-CoA with shikimate to form *p*-coumaroyl shikimic acid. It also catalyzes the reverse reaction converting caffeoyl shikimate ester to caffeoyl-CoA (Hoffmann et al., 2003). Differently, RAS catalyzes the coupling of *p*-coumaroyl-CoA/caffeoyl-CoA and pHPL/DHPL to form 4-coumaroyl-4’-hydroxyphenyllactic acid, 4-coumaroyl-3’,4’-dihydroxyphenyllactic acid, and/or caffeoyl-4’-hydroxyphenyllactic acid in different plant species (**Figure 1**) (Eberle et al., 2009; Petersen et al., 2009; Di et al., 2013; Levsh et al., 2019; Liu et al., 2019; Lu, 2021). Both HCT and RAS belong to the BAHD acyltransferase family and are known as CoA-ester-dependent BAHD hydroxycinnamoyltransferases (Petersen et al., 2009).

**Figure 6:**
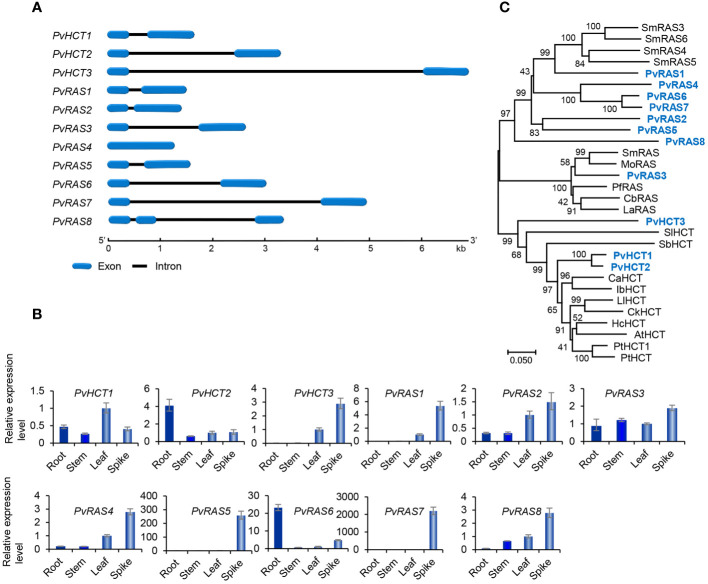
Gene structures, expression patterns and phylogenetic analysis of *PvHCT* and *PvRAS* genes and their deduced proteins. **(A)** The intron-exon structures of three *PvHCT* and *eight PvRAS* genes. **(B)** Fold changes of *PvHCT* and *PvRAS* gene expression in roots, stems, leaves and spikes of *P. vulgaris* plants. The expression level in leaves was arbitrarily set to 1, respectively. **(C)** Phylogenetic analysis of PvHCT and PvRAS proteins. The unrooted Neighbor-Joining tree was constructed using the MEGA program (version 7.0) with default parameters. Proteins included are PvHCT1–PvHCT3, PvRAS1–PvRAS8, and the HCTs and RASs from *S. miltiorrhiza* (Sm), *M. officinalis* (Mo), *P. frutescens* (Pf), *C*. *blumei* (Cb), *L. angustifolia* (La), *Solanum lycopersicum* (Sl), *S. bicolor* (Sb), *C*. *arabica* (Ca), *Ipomoea batatas* (Ib), *L. leucocephala* (Ll), *Caragana korshinskii* (Ck), *Hibiscus cannabinus* (Hc), *Arabidopsis* (At), *P. trichocarpa* (Pt) (**Supplementary Table 2**).

Genome-wide analysis showed that there were three *PvHCT* and eight *PvRAS* genes in *P. vulgaris* (**Table 1**). Except that *PvRAS4* had no intron and *PvRAS8* had two introns, other three *PvHCTs* and six *PvRASs* contained an intron and shared similar gene structures (**Figure 6A**). qRT-PCR analysis showed that the three *PvHCTs* showed differential expression (**Figure 6B**). RNA-seq analysis showed that *PvHCT1* had the highest expression, followed by *PvHCT2* (**Figure 3D**). The expression of *PvHCT3* was very low (**Figure 3D**). It indicates that, among the three *PvHCTs*, *PvHCT1* could be most likely to be involved in RA biosynthesis in *P. vulgaris*. Among the eight *PvRASs*, *PvRAS3* showed the highest expression and was highly expressed in spikes, followed by stems, leaves, and roots (**Figures 3D**, **6B**). The expression of other seven *PvRASs* was relatively low in the tissues analyzed (**Figure 3D**).

Sequence alignment of PvHCT and PvRAS proteins showed that all of them contained the conserved “HXXXD” and “DFGWG” motifs (**Supplementary Figure 8**) (Berger et al., 2006). Phylogenetic analysis of three PvHCTs, eight PvRASs, and HCTs and RASs from various other plants showed that RASs and HCTs separated into two clades (**Figure 6C**). All HCTs were clustered in one clade, whereas all RASs were clustered in the other one. In addition, the RAS clade could be divided into two sub-clades. PvRAS3 was clustered with functionally known RASs from *C. blumei*, *Melissa officinalis*, *Lavandula Angustifolia* and *S. miltiorrhiza* in a sub-clade (Berger et al., 2006; Landmann et al., 2011; Sander and Petersen, 2011; Weitzel and Petersen, 2011; Di et al., 2013; Zhou et al., 2018; Fu et al., 2020). Taken together with the high expression of *PvRAS3* gene (**Figure 6B**), the results suggest the involvement of PvRAS3 in RA biosynthesis. The other RAS sub-clade included PvRAS1, PvRAS2, PvRAS4–PvRAS8, and four putative SmRASs (**Figure 5C**). The function of these RASs is currently unknown. Among them, the expression of *PvRAS2*, *PvRAS4* and *PvRAS8* showed similar patterns with RA distribution (Kim et al., 2014) (**Figure 6B**). It indicates that these *PvRASs* could also be associated with RA biosynthesis.

C3H is the other enzyme involved in downstream of the RA biosynthetic pathway (**Figure 1**). It catalyzes the hydroxylation of *p*-coumaroyl shikimic acid, a shikimate ester of *p*-coumarate generated from *p*-coumaroyl-CoA and shikimate under the catalysis of HCT, into caffeoyl shikimic acid, a shikimate ester of caffeic acid (Schoch et al., 2001). C3H is a cytochrome P450 encoded by members of the *CYP98* gene family (Schoch et al., 2001; Franke et al., 2002; Ralph et al., 2006). Similarily, the enzyme involved in the final step of RA biosynthetic pathway is also a cytochrome P450 encoded by members of the *CYP98* gene family. It introduces the hydroxyl group(s) to the products of RAS (Eberle et al., 2009; Di et al., 2013; Levsh et al., 2019; Liu et al., 2019; Fu et al., 2020) (**Figure 1**).

**Figure 7:**
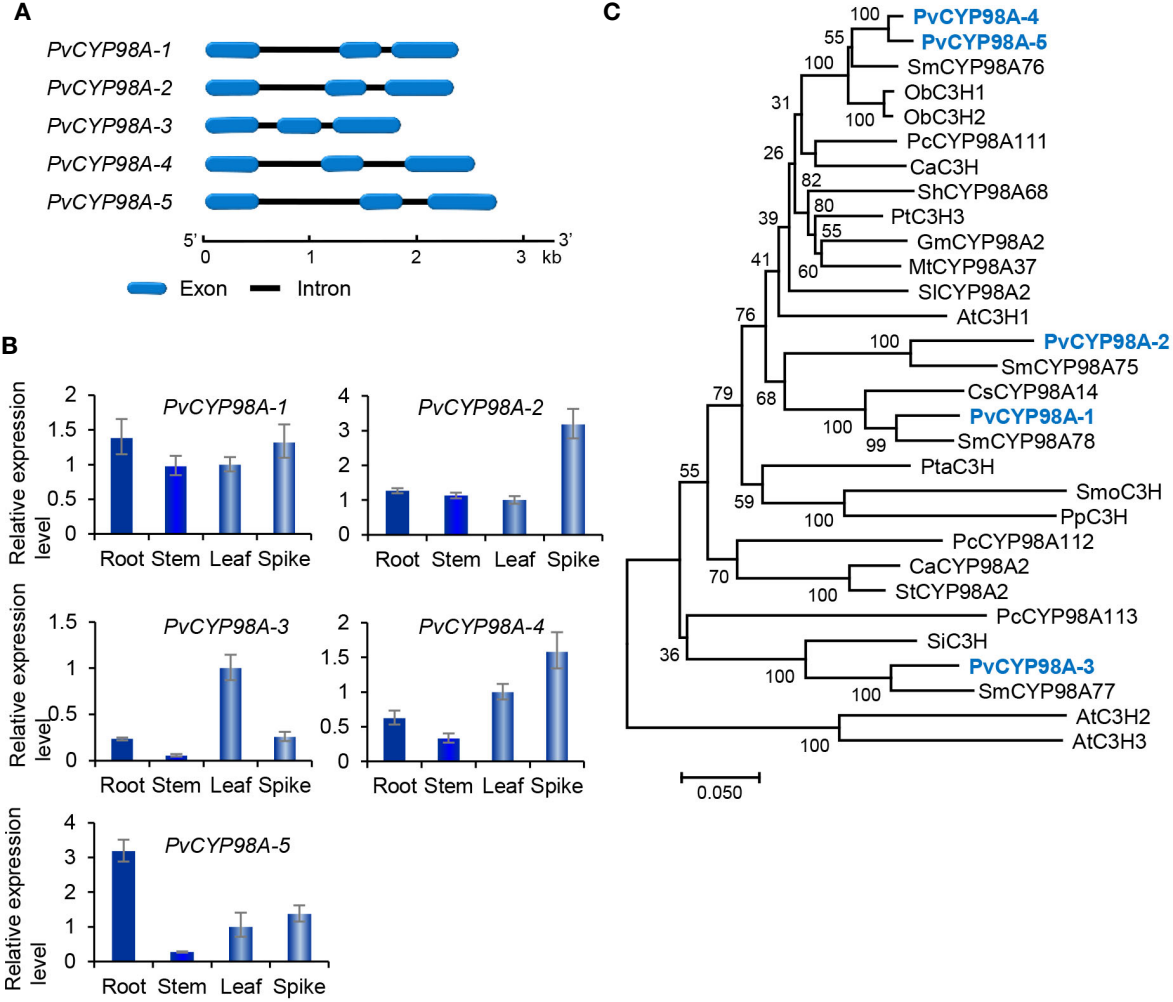
Gene structures, expression patterns and phylogenetic analysis of *PvCYP98* genes and their deduced proteins. **(A)** The intron-exon structures of five *PvCYP98* genes. **(B)** Fold changes of *PvCYP98* gene expression in roots, stems, leaves and spikes of *P. vulgaris* plants. The expression level in leaves was arbitrarily set to 1, respectively. **(C)** Phylogenetic analysis of PvCYP98 proteins. The unrooted Neighbor-Joining tree was constructed using the MEGA program (version 7.0) with default parameters. Proteins included are PvCYP98A-1–PvCYP98A-5 and the C3Hs and CYP98As from *Arabidopsis* (At), *S. miltiorrhiza* (Sm), *Ocimum basilicum* (Ob), *P. campanularia* (Pc), *C*. *arabica* (Ca), *Sinopodophyllum hexandrum* (Sh), *P. trichocarpa* (Pt), **(G)** *max* (Gm), *M. truncatula* (Mt), *S. lycopersicum* (Sl), *C*. *scutellarioides* (Cs), *P. taeda* (Pta), *Selaginella moellendorffii* (Smo), *Physcomitrium patens* (Pp), *Capsicum annuum* (Ca), *S. tuberosum* (St), and *S. indicum* (Si) (**Supplementary Table 2**).

Genome-wide analysis showed that there were five *PvCYP98* genes in *P. vulgaris* (**Table 1**). All of them contain two introns and share similar gene structures (**Figure 7A**). They showed differential expression patterns and *PvCYP98A-1* and *PvCYP98A-2* had relatively high expression among the five *PvCYP98s* (**Figures 3E**, **7B**). Similar to PvC4Hs, the other family of cytochrome P450 proteins involved in RA biosynthesis (**Supplementary Figure 4**), PvCYP98 proteins also contain the five conserved P450 motifs, including “PPGP”, “(A/G)(A/G)X(D/E)T(T/S)”, “EXLR”, “PERF”, and “FGXGRRXCXG” (**Supplementary Figure 9**) (Eberle et al., 2009; Khatri et al., 2023). Phylogenetic analysis showed that PvCYP98A-4 and PvCYP98A-5 were clustered with the functionally known C3Hs (**Figure 7C**), such as AtC3H1 (AtCYP98A3) and PtC3H3 (Schoch et al., 2001; Chen et al., 2011). PvCYP98A-1 and PvCYP98A-2 were clustered with CsCYP98A14 and SmCYP98A78 involved in the last step of RA biosynthetic pathway (**Figure 7C**) (Eberle et al., 2009; Di et al., 2013; Liu et al., 2019; Fu et al., 2020). PvCYP98A-3 was clustered with the putative *Sesamum indicum* SiC3H and *P. campanularia* PcCYP98A113 involved in the last step of RA biosynthetic pathway (**Figure 6C**) (Anterola et al., 2002; Levsh et al., 2019). Taken together with the expression patterns of *PvCYP98As* (**Figures 3E**, **7B**) and the distribution patterns of RAs in *P. vulgaris* (Kim et al., 2014), the results indicated that PvCYP98A-1 and PvCYP98A-2 could be involved in the hydroxylation of RAS products, PvCYP98A-4 and PvCYP98A-5 could be PvC3Hs, whereas the function of PvCYP98A-3 remained to be analyzed.

### PvRAS3 and PvRAS4 were involved in RA biosynthesis *in vitro*

**Figure 8:**
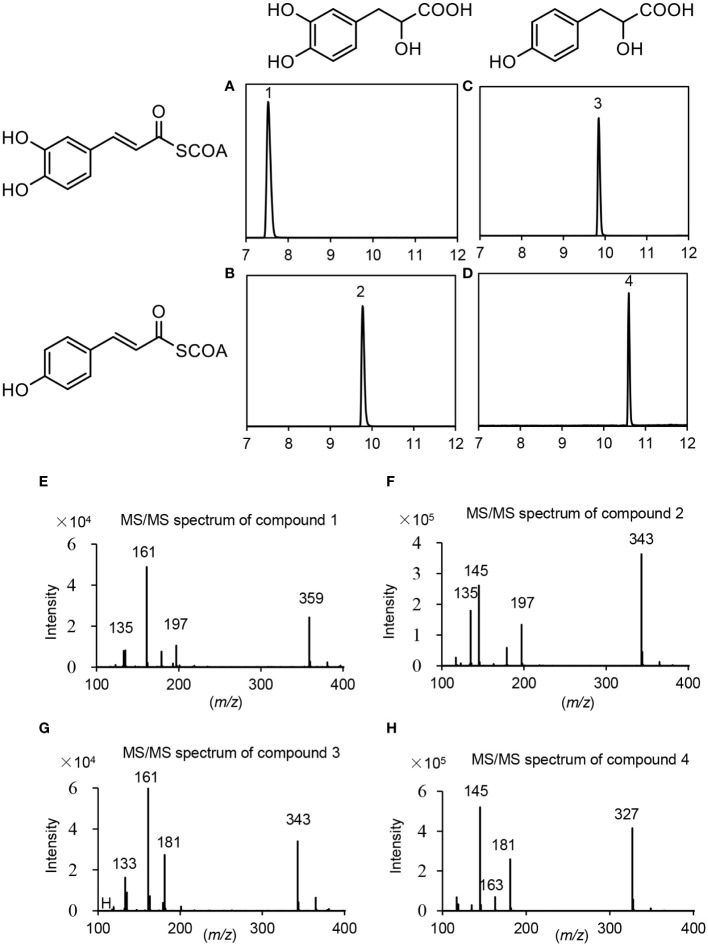
UPLC and LC-MS/MS detection of *in vitro* reaction products catalyzed by PvRAS3. **(A–D)** UPLC chromatograms of the reaction products catalyzed by PvRAS3 using caffeoyl-CoA and DHPL **(A)**, *p*-coumaroyl-CoA and DHPL **(B)**, caffeoyl-CoA and pHPL **(C)**, and *p*-coumaroyl-CoA and pHPL **(D)** as acyl donors and acceptors, respectively. **(E–H)** MS/MS spectra of compounds 1, 2, 3, and 4.

Based on gene expression patterns, phylogenetic relationships and the significance of RAS in RA biosynthesis, *PvRAS3* and *PvRAS4* were selected for functional analysis using experimental approaches. Among them, *PvRAS3* showed the highest expression among the eight *PvRAS* genes identified and was clustered with functionally known RASs in the phylogenetic tree constructed (**Figures 6B, C**). *PvRAS4* was one of the three *PvRAS* genes with expression patterns similar to RA distribution patterns (Kim et al., 2014) (**Figure 6B**). *PvRAS3* and *PvRAS4* cDNAs were cloned and introduced into *E. coli* competent cells. Recombinant proteins were induced and purified (**Supplementary Figure 10**). To assay the activity of recombinant PvRAS3 enzyme, *p*-coumaroyl-CoA or caffeoyl-CoA was used as the acyl donor, and pHPL or DHPL was used as the acyl acceptor. Negative controls were performed with *E. coli* BL21 (DE3) cells transformed with the empty pGEX-4T-1 vector (**Supplementary Figure 11**). LC-MS/MS analysis showed that PvRAS3 could catalyze the condensation of acyl donors and acceptors to generate four compounds, respectively (**Figures 8A–D**). Compound 1, generated through the condensation of caffeoyl-CoA and DHPL, was identified as RA based on UPLC and LC-MS/MS analyses (**Figures 8A, E**) and previous publication (Landmann et al., 2011; Di et al., 2013). Compounds 2 (**Figure 8B**) and 3 (**Figure 8C**) had the formula C_18_H_16_O_7_ according to the MS spectra in negative mode [M-H]^–^ (*m/z* = 343) (**Figures 8F, G**). They corresponded to an ester of caffeoyl-CoA and DHPL (caffeoyl-4’-hydroxyphenyllactic acid) or *p*-coumaroyl-CoA and pHPL (4-coumaroyl-3’,4’-dihydroxyphenyllactic acid), respectively, as described previously (Landmann et al., 2011). Compound 4 was determined to be the ester of *p*-coumaroyl-CoA and pHPL based on the negative ion spectra (*m/z* = 327, C_18_H_15_O_6_) (**Figures 8D, H**) and previous publication (Landmann et al., 2011).

For kinetic analysis of PvRAS3, *p*-coumaroyl-CoA or caffeoyl-CoA was used as the acyl donor and pHPL or DHPL was used as the acyl acceptor. To test the acceptor specificity, the concentration of the donor substrates remained saturated, while the levels of the acceptor substrates were varied (**Supplementary Figure 12**). When caffeoyl-CoA was used as the donor, the *K*m values of PvRAS3 with DHPL and pHPL were 197.6 and 166.8 μM, respectively (**Table 2**). Using *p*-coumaroyl-CoA as the donor, the *K*m values of DHPL and pHPL were 41.7 and 32.1 μM, respectively. The results suggested that the *K*m values of DHPL and pHPL with *p*-coumaroyl-CoA were lower than those with caffeoyl-CoA. To assess the donor affinity, the concentrations of the acceptors (DHPL and pHPL) were kept constant while the levels of caffeoyl-CoA and *p*-coumaroyl-CoA were varied. The results showed that the *K*m value of PvRAS3 for Caffeoyl-CoA was approximately 5.7-fold higher than that for *p*-coumaroyl-CoA (**Table 2**). Overall, PvRAS3 exhibited a high affinity toward DHPL and pHPL when *p*-coumaroyl-CoA was used as the acyl donor.

**Table 2:** Kinetic parameters of recombinant PvRAS3 toward different substrates.

Saturating subatrate	Varying substrate	V_max_ (nkat mg^-1^)	K_m_ (µM)	K_cat_ (S^-1^)	K_cat_/*K*_m_ (M^-1^ S^-1^)
Caffeoyl-CoA	DHPL	187.6 ± 1.22	197.6 ± 3.4	14.0 ± 1.2	0.7×10^5^
*C*affeoyl-CoA	pHPL	189.7 ± 9.3	166.8 ± 14.4	13.2± 1.4	0.8 ×10^5^
*p*-coumaroyl-CoA	DHPL	303.0 ± 5.0	41.7 ± 1.2	30.5 ± 2.1	7.0 ×10^5^
*p*-coumaroyl-CoA+	pHPL	323.4 ± 5.4	32.1 ± 0.7	33.3 ± 2.3	10.0 ×10^5^
DHPL	Caffeoyl-CoA	165.8 ± 6.2	173.2 ± 9.0	14.1 ± 1.0	0.8×10^5^
DHPL	*p*-coumaroyl-CoA	307.5 ± 4.0	28.9 ± 7.5	13.1 ± 1.1	5.0 ×10^5^
pHPL	Caffeoyl-CoA	155.6 ± 6.3	175.0 ± 9.1	32.8 ± 2.1	1.9 ×10^5^
pHPL	*p*-coumaroyl-CoA	311.5 ± 3.2	30.6 ± 1.2	34.3 ± 2.2	11.0 ×10^5^

**Figure 9:**
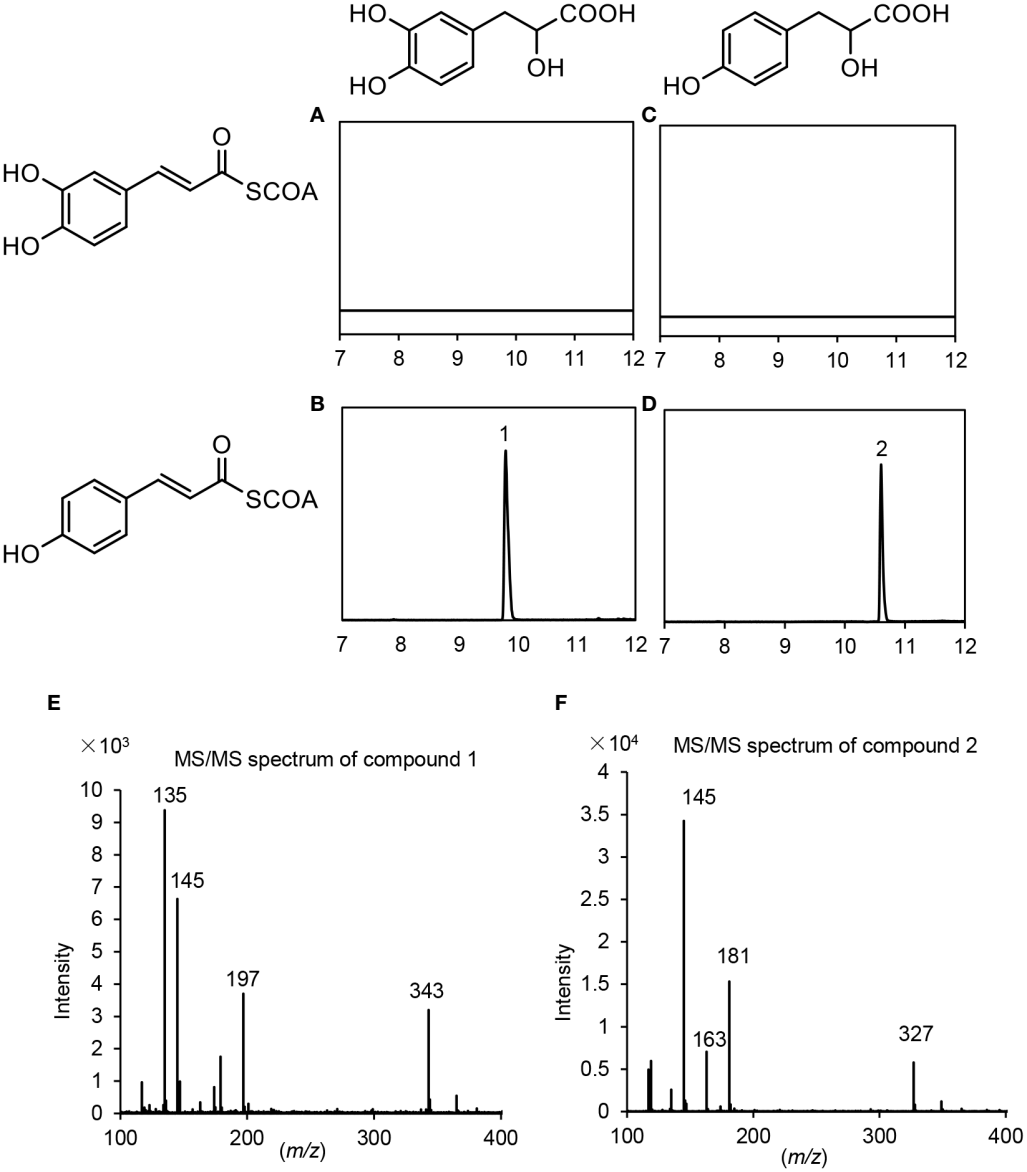
UPLC and LC-MS/MS detection of *in vitro* reaction products catalyzed by PvRAS4. **(A–D)** UPLC chromatograms of the reaction products catalyzed by PvRAS4 using caffeoyl-CoA and DHPL **(A)**, *p*-coumaroyl-CoA and DHPL **(B)**, caffeoyl-CoA and pHPL **(C)**, and *p*-coumaroyl-CoA and pHPL **(D)** as acyl donors and acceptors, respectively. **(E, F)** MS/MS spectra of compounds 1 and 2.

Similarly, *p*-coumaroyl-CoA, caffeoyl-CoA, pHPL and DHPL were also used as substrates for the analysis of PvRAS4 (**Supplementary Figure 13**). LC-MS/MS analysis showed that products could be detected when *p*-coumaroyl-CoA was used as acyl donor and pHPL or DHPL were used as acyl acceptor (**Figures 9B, D**). Based on MS spectra, the products were identical to compounds 2 and 4 catalyzed by PvRAS3 (**Figures 9E, F**). The *K*_cat_/*K*_m_ values of PvRAS4 for *p*-coumaroyl-CoA and pHPL or DHPL were smaller than those of PvRAS3 (**Table 3**). No product was found when caffeoyl-CoA was used as acyl donor (**Figures 9A, B**). The results indicated that PvRAS4 could use *p*-coumaroyl-CoA, but not caffeoyl-CoA, as acyl donor. However, its affinity toward *p*-coumaroyl-CoA was lower than PvRAS3.

**Table 3:** Kinetic parameters of recombinant PvRAS4 toward different substrates.

Saturating subatrate	Varying substrate	V_max_ (nkat mg^-1^)	K_m_ (µM)	K_cat_ (S^-1^)	K_cat_/*K*_m_ (M^-1^ S^-1^)
Caffeoyl-CoA	DHPL	N.D.	N.D.	N.D.	N.D.
*C*affeoyl-CoA	pHPL	N.D.	N.D.	N.D.	N.D.
*p*-coumaroyl-CoA	DHPL	122.6± 5.7	226.9 ± 17.5	4.3± 1.2	0.2×10^5^
*p*-coumaroyl-CoA+	pHPL	125.5 ± 3.6	228.5 ± 16.6	5.1 ± 2.1	0.2 ×10^5^
DHPL	Caffeoyl-CoA	N.D.	N.D.	N.D.	N.D.
DHPL	*p*-coumaroyl-CoA	132.5 ± 9.2	217.5 ± 10.3	4.3 ± 1.6	0.2×10^5^
pHPL	Caffeoyl-CoA	N.D.	N.D.	N.D.	N.D.
pHPL	*p*-coumaroyl-CoA	127.0 ± 5.9	216.8 ± 15.2	5.4± 1.3	0.2×10^5^

N.D., not detected.

### Existence of RA, 4-coumaroyl-3’,4’-dihydroxyphenyllactic acid, 4-coumaroyl-4’-hydroxyphenyllactic acid and caffeoyl-4’-hydroxyphenyllactic acid in *P. vulgaris* plants

**Figure 10:**
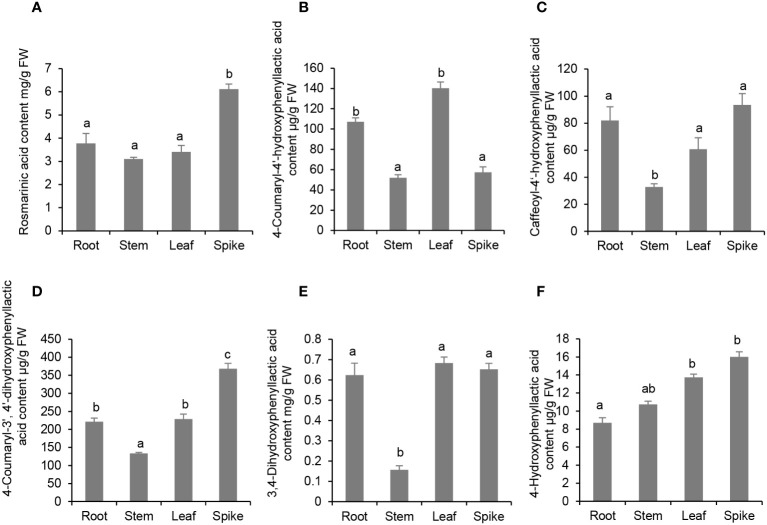
Analysis of phenolic acid contents in roots, stems, leaves and spikes of *P. vulgaris*. **(A)** Rosmarinic acid; **(B)** 4-Coumaroyl-4’-hydroxyphenyllactic acid; **(C)** Caffeoyl-4’-hydroxyphenyllactic acid; **(D)** 4-Coumaroyl-3’, 4’-dihydroxyphenyllactic acid; **(E)** 3,4-Dihydroxyphenyllactic acid; **(F)** 4-Hydroxyphenyllactic acid. Error bars represent standard deviations of mean value from three biological replicates. ANOVA (analysis of variance) was calculated using SPSS. *P* < 0.05 was considered statistically significant and was represented by different letters above the bars. *P* ≥ 0.05 was considered statistically non-significant and was represented by same letters above the bars.

Enzyme activity assay showed that PvRAS3 could condense *p*-coumaroyl-CoA and caffeoyl-CoA with pHPL and DHPL *in vitro*. To analyze whether the products exist in *P. vulgaris* plants, phenolic acid compounds were extracted from roots, stems, leaves and flowers and analyzed using UPLC and LC-MS/MS as described (Kim et al., 2014). The results showed that all of the four products, including RA, 4-coumaroyl-3’,4’-dihydroxyphenyllactic acid, 4-coumaroyl-4’-hydroxyphenyllactic acid and caffeoyl-4’-hydroxyphenyllactic acid, could be detected in the tissues analyzed (**Figure 10**; **Supplementary Figure 14**). RA was highly accumulated at the level of mg g^-1^ fresh weight (FW) with the highest level of 6.1 mg g^-1^ FW in flowers and the lowest level of 3.1 mg g^-1^ FW in stems (**Figure 10A**). The contents of 4-coumaroyl-4’-hydroxyphenyllactic acid ranged from 51.9 µg g^-1^ FW in roots to 140.3 µg g^-1^ FW in leaves (**Figure 10B**). The contents of 4-coumaroyl-4’-hydroxyphenyllactic acid were relatively low, which ranged from 32.9 µg g^-1^ FW in roots to 93.5 µg g^-1^ FW in flowers (**Figure 10C**). The contents of 4-coumaroyl-3’,4’-dihydroxyphenyllactic acid ranged from 133.3 µg g^-1^ FW in roots to 368.0 µg g^-1^ FW in flowers (**Figure 10D**). The contents of RA and 4-coumaroyl-3’,4’-dihydroxyphenyllactic acid in *P. vulgaris* were higher but comparable to those in lavender flowers, which were 2 mg g^-1^ FW and 150 µg g^-1^ FW, respectively (Landmann et al., 2011). To our best knowledge, this is the first report to detect 4-coumaroyl-4’-hydroxyphenyllactic acid and caffeoyl-4’-hydroxyphenyllactic acid in plants. In addition, we also analyzed the contents of 3,4-dihydroxyphenyllactic acid and 4-hydroxyphenyllactic acid. Among them, 3,4-dihydroxyphenyllactic acid was highly accumulated in roots, leaves and flowers (**Figure 10E**), whereas the content of 4-hydroxyphenyllactic acid was relatively higher in leaves and flowers than roots and stems (**Figure 10F**). The results suggest that both the substrates and products of the four reactions catalyzed by PvRAS3 and/or PvRAS4 *in vitro* exist in *P. vulgaris* plants.

### CRISPR/Cas9-mediated functional analysis of PvRAS3 in *P. vulgaris* hairy roots

**Figure 11:**
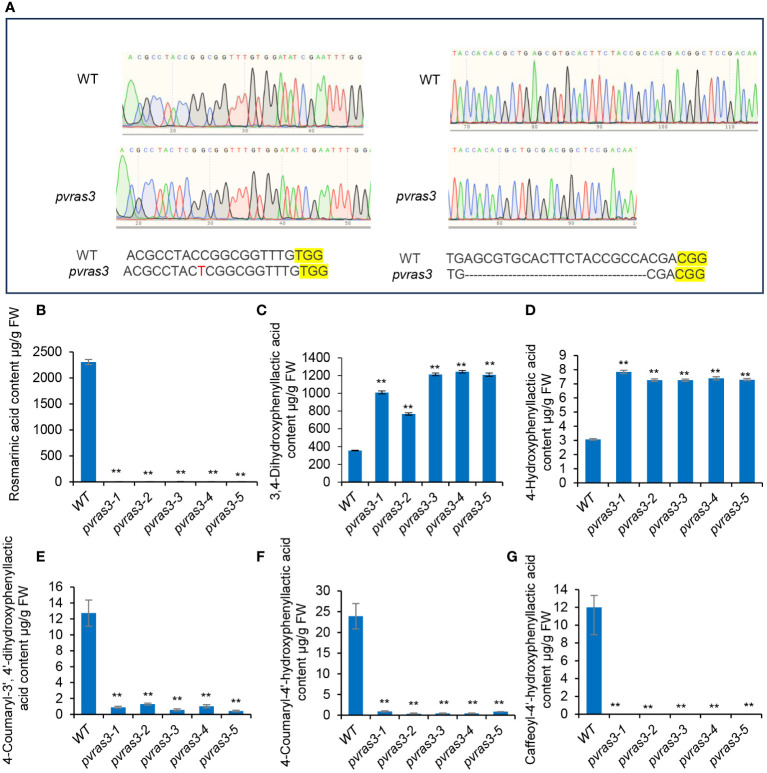
Analysis of phenolic acid contents in hairy roots of wild type and *pvras3* mutants. **(A)** The sgRNA-edited nucleotide sequences in *pvras3* mutants. WT, wild type. **(B–G)** Contents of rosmarinic acid **(B)**, 3,4-dihydroxyphenyllactic acid **(C)**, 4-hydroxyphenyllactic acid **(D)**, 4-coumaroyl-3’,4’-dihydroxyphenyllactic acid **(E)**, 4-coumaroyl-4’-hydroxyphenyllactic acid **(F)** and caffeoyl-4’-hydroxyphenyllactic acid **(G)** in wild type (WT) and five *pvras3* mutant lines. *P* < 0.05 (*) and *P* < 0.01 (**) were considered statistically significant and highly significant, respectively.

Gene expression profiling and *In vitro* enzyme activity assay showed that PvRAS3 could be the main RAS catalyzing RA biosynthesis in *P. vulgaris*. To gain further insight into the involvement of PvRAS3 in RA biosynthesis, we designed two guide RNAs (gRNAs) targeting the first coding exon of *PvRAS3* for the CRISPR/Cas9 gene-editing tool. Five lines of transgenic hairy roots with the same insertion and deletion patterns in *PvRAS3* gene were obtained (**Figure 11A**). It indicated that these transgenic lines were homozygous mutants of *PvRAS3*, hereinafter referred to as *pvras3-1*, *pvras3-2*, *pvras3-3*, *pvras3-4* and *pvras3-5*, respectively. Analysis of RA, 4-coumaroyl-3’,4’-dihydroxyphenyllactic acid, 4-coumaroyl-4’-hydroxyphenyllactic acid and caffeoyl-4’-hydroxyphenyllactic acid showed that the contents of these compounds decreased significantly in *pvras3* mutants with the contents of RA and caffeoyl-4’-hydroxyphenyllactic acid almost below the detection limit (**Figures 11B, E–G**). On the contrary, the contents of DHPL and pHPL increased significantly in the mutants (**Figures 10C, D**). It confirmed the catalytical function of PvRAS3 and its significance in RA biosynthesis.

## Conclusions

*P. vulgaris* is a species of the Lamiaceae family with significant medicinal value. RA is one of the major bioactive components in *P. vulgaris* medicinal materials. Through genome-wide analysis, a total of 51 P*. vulgaris* genes belonging to seven RA biosynthesis-related gene families were identified. Subsequent gene and protein feature analysis, gene expression analysis and phylogenetic relationship analysis showed that seventeen of them, including *PvPAL1*, *PvPAL2*, *PvC4H1*, *PvC4H2*, *Pv4CL1*, *Pv4CL3*, *Pv4CL6*, *Pv4CL7*, *Pv4CL8*, *PvTAT3*, *PvTAT4*, *PvHPPR1*, *PvHPPR3*, *PvRAS3*, *PvRAS4*, *PvCYP98A-1* and *PvCYP98A-2*, could be involved in RA biosynthesis. *In vitro* enzymatic assay showed that both of PvRAS3 and PvRAS4 were involved in RA biosynthesis. PvRAS3 could catalyze the condensation of *p*-coumaroyl-CoA and caffeoyl-CoA with pHPL and DHPL. The affinity of PvRAS3 toward *p*-coumaroyl-CoA was higher than caffeoyl-CoA. PvRAS4 only catalyzed the condensation of *p*-coumaroyl-CoA with pHPL and DHPL. The affinity of PvRAS4 toward *p*-coumaroyl-CoA was lower than PvRAS3. These results were consistent with *in vivo* phenolic acid compound determination and *PvRAS3* transgenic analysis. UPLC analysis of phenolic acid compounds showed the existence of RA, 4-coumaroyl-3’,4’-dihydroxyphenyllactic acid, 4-coumaroyl-4’-hydroxyphenyllactic acid and caffeoyl-4’-hydroxyphenyllactic acid in roots, stems, leaves and flowers of *P. vulgaris*. Generation of *pvras3* homozygous mutants through CRISPR/Cas9 technology and subsequent chemical compound analysis showed that the contents of RA, 4-coumaroyl-3’,4’-dihydroxyphenyllactic acid, 4-coumaroyl-4’-hydroxyphenyllactic acid and caffeoyl-4’-hydroxyphenyllactic acid decreased significantly, whereas the contents of DHPL and pHPL increased significantly in *pvras3* mutants. These results indicate the existence of four possible RA biosynthetic routes in *P. vulgaris*, which remains to be further confirmed through the analysis of *PvCYP98A* genes (**Figure 1**). Among them, routes 1 and 2 could be the main routes. PvRAS3 was the main enzyme catalyzing the condensation of acyl donors and acyl acceptors during RA biosynthesis in *P. vulgaris*. PvRAS4 could play a minor role. Further functional analysis of other fourteen candidate genes, particularly *PvCYP98A-1* and *PvCYP98A-2*, may provide a more complete picture of RA biosynthetic pathway.

## Data availability statement

The original contributions presented in the study are included in the article/**Supplementary Material**. Further inquiries can be directed to the corresponding authors.

## Author contributions

CY: Data curation, Writing – original draft. CL: Data curation, Methodology, Validation, Writing – review & editing. MJ: Data curation, Writing – review & editing. YX: Data curation, Writing – review & editing. SZ: Writing – review & editing. XH: Writing – review & editing. YC: Writing – review & editing. SL: Methodology, Software, Supervision, Writing – original draft, Writing – review & editing.
