# Supplementary material for: Systematic characterization of gene families and functional analysis of PvRAS3 and PvRAS4 involved in rosmarinic acid biosynthesis in Prunella vulgaris
Source: Front Plant Sci. 2024 May 1;15:1374912. doi: 10.3389/fpls.2024.1374912 (PMC11094360; doi:10.3389/fpls.2024.1374912)
Supplement: Supplementary file 1 [file DataSheet_1.pdf]

Supplementary data for

Systematic characterization of gene families and functional analysis of *PvRAS3* and *PvRAS4* involved in rosmarinic acid biosynthesis in *Prunella vulgaris*

Chao Yan [1,2,3,†], Caili Li [1,2,†], Maochang Jiang [1,2], Sixuan Zhang [1,2], Xiangling Hu [3], Yuhang Chen [3,*] and Shanfa Lu [1,2,*]

[1] State Key Laboratory for Quality Ensurance and Sustainable Use of Dao-di Herbs, Institute of Medicinal Plant Development, Chinese Academy of Medical Sciences & Peking Union Medical College, Beijing 100193, China

[2] Engineering Research Center of Chinese Medicine Resource, Ministry of Education, Institute of Medicinal Plant Development, Chinese Academy of Medical Sciences & Peking Union Medical College, Beijing 100193, China

[3] College of Pharmaceutical Sciences, Chengdu Medical College, Chengdu 610500, China

Table S1. Primers used for PCR and gene cloning.

**Primers**	**Sequnence （5’ to 3’）**
Primers used for RT-qPCR	
PvPAL1-F	CCGGAATCCTAGCCTTGACT
PvPAL1-R	TCTTGATTGTGCTGCTCTGC
PvPAL2-F	CGACCTTGTTCCACTCTCCT
PvPAL2-R	GACTCCGACGAGCTTGAATG
PvPAL3-F	AGAAGGAGCTGAGCAGTTGT
PvPAL3-R	TGTTCTCGATCGACGGCTTA
PvPAL4-F	CAACCCGCTGATCGATGTTT
PvPAL4-R	GGCCAACCTCGAGTTATCCA
PvPAL1-R	TCTTGATTGTGCTGCTCTGC
PvC4H1-F	TTCGACATCCCTGCTGAGAG
PvC4H1-R	ACCTGAAGTCATTGCCGTTG
PvC4H2-F	CAATGCATGGTGGTTAGCGA
PvC4H2-R	TCGCCTCAACTTTAGCCTCT
PvC4H3-F	TACGACATACCGAGCGAGAG
PvC4H3-R	GGTGGCTTCCACCTTAGACT
Pv4CL1-F	GGTTCATTGATGGCGACGAC
Pv4CL1-R	GCGTCAGAGATGTGTGGATG
Pv4CL2-F	ATAGTGGTGGCGATGGTGAA
Pv4CL2-R	CCCGGCAATTTCTTGGTGAA
Pv4CL3-F	ATCTGCATTCGTGGTCCTCA
Pv4CL3-R	GTCGTCATCCACGAACCCTA
Pv4CL4-F	AAGTTCCTCCGGCAGAATTG
Pv4CL4-R	ACTTATAGTGGTGCCCGCTT
Pv4CL5-F	AATGGAGCTCATCGCCAAAC
Pv4CL5-R	TGAACCAACTCCCTCCTCAC
Pv4CL6-F	GGATTGTGGACGATGACGAC
Pv4CL6-R	GTACAACGGCAGCATCAGAA
Pv4CL7-F	GCGGTCAGAGACAAGTTTCC
Pv4CL7-R	GCCAAACACATTGCCAACAC
Pv4CL8-F	GCTGACTGCAAGAAGAGTCG
Pv4CL8-R	CCGAGGAACGAGAAGACGTA
Pv4CL9-F	CGTACTTCAGTGCCAAGGTG
Pv4CL9-R	TTCATCACACTCGGGCTCTT
Pv4CL10-F	ATTTCAGGAATGCCCAAGCC
Pv4CL10-R	CTTAATGCGGTCGACAACGA
Pv4CL11-F	GAGATTCTCCGCCTCCTACC
Pv4CL11-R	GCTGCTCAGATCGTACTTGC
Pv4CL12-F	AGCATTTGCTGCCTTTGCTA
Pv4CL12-R	ATCTACAACCTCCGGAACCC
Pv4CL13-F	TCCACGTGTACGGCTTGATA

Pv4CL13-R	ATCTCCATCACCTCGTGGAC
Pv4CL14-F	GTGCCTGTTCTCGTCACTTC
Pv4CL14-R	CCGATTGATTCGGCTTCTCC
Pv4CL15-F	GGATGAAGAAGCGGGAGAGA
Pv4CL15-R	GTTCATGACAGCAGCCTCAG
Pv4CL16-F	GGTATGACAGAAGCGGGAGT
Pv4CL16-R	AGTTCCACATGAACCAGCCT
Pv4CL17-F	GCAGGGTGACGATTGCGCCGTT
Pv4CL17-R	AGGGTGCGGCACCGCAGGTT
PvTAT1-F	TCGAGGCTCGCTACTTTGAT
PvTAT1-R	CCGGGTTTATGACGACGATG
PvTAT2-F	GTCTCTGTCCTGGAAGGCAT
PvTAT2-R	ATACCCTGGGAGGAGTACGA
PvTAT3-F	ATTGCTGTGGGTCTAAAGAA
PvTAT3-R	CAGAGCAGATGGCTCCACTGCAA
PvTAT4-F	ATCGCTGTGGGTCTCAAGAA
PvTAT4-R	AAGAGCAGATGGCTCGACTGCGA
PvTAT5-F	GGAACGAGCGTGTTATTGGG
PvTAT5-R	CGCCTTGGCTCAAATGAATG
PvTAT6-F	GAGGCAATGGGAGTGTTTGG
PvTAT6-R	AAGCAGCAATCCAACCAAGG
PvTAT7-F	CACAGCTGATCCTGCAACTC
PvTAT7-R	ACATGCATCGGCAGATTCTC
PvHPPR1-F	TGATGAGGCTGAACTGGTGT
PvHPPR1-R	CCTACATGTGGCAAGAGCAC
PvHPPR2-F	GAGCGGATCGTGGAGAAATG
PvHPPR2-R	CCAAACGCCTTTGCTCTTCT
PvHPPR3-F	AGCGACATCCTAGTGGTAGC
PvHPPR3-R	GCCCAACGCATCAATCACTT
PvHPPR4-F	ACCTACTTGCGGGACTTGAT
PvHPPR4-R	TTTGCAATGACGAGGTCTGC
PvHCT1-F	TGGTGCAGCCGAGACGGTAGTC
PvHCT1-R	TTGCCCTCATCCTTGGACTT
PvHCT2-F	CGGTGGATTACTCGAAGGGA
PvHCT2-R	AAGACCGGAGAATCCGTCAG
PvHCT3-F	CCTCAAGTACTCGTCGTCCA
PvHCT3-R	GGGTTGTGTTACGTGTTGGG
PvRAS1-F	GGATTCTTCGGCAACGGAAT
PvRAS1-R	CCTCAAGTAGTCGTCGTCCA
PvRAS2-F	GCATGAGGCGATAGCACAAA
PvRAS2-R	CCAAATTCGGGCACTTGACT
PvRAS3-F	CGCCATTTGAAGGCAAGAGT
PvRAS3-R	GGTCCATGTGTTGGGTATGC
PvRAS4-F	CGGCGAAATTGAGACGAAAC

PvRAS4-R	ACGTAATCAAGAGCCGACCT
PvRAS5-F	TAAGGGCCTTTCGACCTACC
PvRAS5-R	GCGAATAGGAGACCGGATGA
PvRAS6-F	AGAGCTGAGATCTCCTTCGC
PvRAS6-R	CTAACTCCGCCGCATTTGAA
PvRAS7-F	CGCTTCCACAGCACTACTTC
PvRAS7-R	CCTCATGTACTCGTCGTCCA
PvRAS8-F	GCTACTACGGCAACGTCATC
PvRAS8-R	CCTCAAGTACTCGTCGTCCA
PvCYP98A-1-F	TGTTCTCACCGAAGAGGCTT
PvCYP98A-1-R	GAATGCCATTGATGCGAGGT
PvCYP98A-2-F	TGGATGTTCCCACTGAACGA
PvCYP98A-2-R	TGTGAGCAACGCATCACAAA
PvCYP98A-3-F	TTCTTGTTCAAGGAACAAAAC
PvCYP98A-3-R	GGTGAGCAAAGCGTCGACGAA
PvCYP98A-4-F	GCGCCTCCCAACGGGATGAGTA
PvCYP98A-4-R	CCGTAGCGATACGCTTGTACAG
PvCYP98A-5-F	ATCCTCCCAAGGGAATGAGAAC
PvCYP98A-5-R	CTGCAGTGATACGCTTGTAAAG
eIF-2-F	TTTTGGGAGAGCGGACACAA
eIF-2-R	AGCTGCCTTGGAGACTGAAA
Primers used for gene cloning	
RAS3-F	ATGAAGATCGAAGTGAAGGAGT
RAS3-R	GATGTCATAAAAGAAGTTTTGA
RAS4-F	ATGAAGATCACAGTGAAGGAGA
RAS4-R	AATATCGTAAAGAAGCTTCTGG

Table S2. Proteins used for phylogenetic analysis.

Plant species	Protein names	Protein IDs
Salvia miltiorrhiza	SmPAL1	ABR14606.1
	SmPAL2	full length of ACT22906.1
	SmPAL3	AGW27206.1
Arabidopsis thaliana	AtPAL1	At2g37040
	AtPAL2	At3g53260
	AtPAL3	At5g04230
	AtPAL4	At3g10340
Populus trichocarpa	PtPAL1	ACC63888.1
	PtPAL2	ACC63890.1
	PtPAL3	ACC63887.1
	PtPAL4	ACC63891.1
	PtPAL5	ACC63889.1
Melissa officinalis	MoPAL	CBJ23826.1
Oryza sativa	OsPAL	BAD23155.1
Zea mays	ZmPAL	NP 001147433.2
Salvia miltiorrhiza	SmC4H1	ABC75596.1
Coffea Arabica	CaC4H4	AFP49808.1
Populus trichocarpa	PtC4H1	ACC63873.1
	PtC4H2	ACC63871.1
	PtC4H3	ACC63872.1
Solanum tuberosum	StC4H	ABC69046.1
Gossypium hirsutum	GhC4H	ACZ06239.1
Glycine max	GmC4H	ACR44227.1
Leucaena leucocephala	LlC4H	AEM63594.1
Scutellaria baicalensis	SbC4H	ADN32769.1
Zinnia elegans	ZeC4H	AAB42024.1
Helianthus tuberosus	HtC4H	CAA78982.1
Helichrysum aureonitens	HaC4H	AFV47361.1
Arabidopsis	AtC4H	At2g30490
Pinus taeda	PtaC4H	AAD23378.1
Anthoceros agrestis	AaC4H	QHF16161.1
Sorghum bicolor	SbiC4H	AAK54447.1
Oryza sativa	OsC4H	AAV44089.1
Erythranthe guttata	EgC4H	EYU36905.1
Salvia miltiorrhiza	Sm4CL1	AAP68990.1
	Sm4CL2	AAP68991.1
	Sm4CL3	AGW27193.1
	Sm4CL4	AGW27194.1
	Sm4CL5	AGW27195.1
Arabidopsis thaliana	At4CL1	At1g51680

	At4CL2	At3g21240
	At4CL3	At1g65060
	At4CL4	At3g21230
	At4CL-like1	At1g62940
	At4CL-like2	At1g20480
	At4CL-like3	At1g20490
	At4CL-like4	At1g20500
	At4CL-like5	At1g20510
	At4CL-like6	At4g19010
	At4CL-like7	At4g05160
	At4CL-like8	At5g38120
	At4CL-like9	At5g63380
Oryza sativa	Os4CL1	Q0DV32.2
	Os4CL2	Q336M7.3
	Os4CL3	Q6YYZ2.1
	Os4CL4	Q10S72.1
	Os4CL5	Q7F1X5.1
	Os4CL6	Q8RU95.2
	Os4CL7	Q69RG7.1
	Os4CL8	Q8GVF9.1
	Os4CL9	Q7XXL2.2
Melissa officinalis	Mo4CL1	CBJ23825.1
Populus trichocarpa	Pt4CL3	ACC63867.1
Salvia miltiorrhiza	SmTAT1	ABC60050.1
	SmTAT2	AGW27212.1
	SmTAT3	AGW27192.1
Arabidopsis thaliana	AtTAT1	AT5G53970.1
	AtTAT2	AT5G36160.1
	AtTAT3	AT2G24850.1
	AtTAT4	At4G23590.1
	AtTAT5	AT4G28420.2
	AtCORI3	AT4G23600.1
	AtRSA1	AT4G28410.1
	AtSUR1	AT2G20610.1
Perilla frutescens	PfTAT	ADO17550.1
Coleus scutellarioides	CsTAT	CAD30341.1
Scutellaria baicalensis	SbTAT1	AIV98132.1
	SbTAT2	AIV98133.1
Solanum pennellii	SpTAT1	ADZ24702.1
Glycine max	GmTAT	NP_001238408.1
Medicago truncatula	MtTAT	AAY85183.1
Salvia miltiorrhiza	SmHPPR1	AAZ67354.1
	SmHPPR2	AGW27202.1
	SmHPPR3	AGW27203.1

Arabidopsis thaliana	AtHPPR2	At1g79870
	AtHPPR3	At1g12550
	AtHPPR4	At2g45630
Agastache rugosa	ArHPPR	QOC77911.1
Mentha aquatica	MaHPPR	AVZ47167.1
Melissa officinalis	MoHPPR	QQW37724.1
Coleus scutellarioides	CsHPPR	CAD47810.2
Scutellaria baicalensis	SbHPPR	AHB63232.1
Sesamum indicum	SiHPPR	XP_011072370.1
Nicotiana tomentosiformis	NtHPPR	XP_009592777.1
Arachis hypogaea	AhHPPR	XP_025617663.1
Zea mays	ZmHPPR	NP_001150094.1
Oryza sativa	OsHPPR	XP_015616725.1
Nymphaea colorata	NcHPPR	XP_031487301.1
Salvia miltiorrhiza	SmRAS	ADA60182.1
	SmRAS3	AGW27208.1
	SmRAS4	AGW27209.1
	SmRAS5	AGW27210.1
	SmRAS6	AGW27211.1
Melissa officinalis	MoRAS	G0LD36.1
Perilla frutescens	PfRAS	AGH61997.1
Coleus blumei	CbRAS	A0PDV5.1
Lavandula Angustifolia	LaRAS	AEA36976.1
Solanum lycopersicum	SlHCT	CAE46933.1
Sorghum bicolor	SbHCT	XP_002452435.1
Coffea Arabica	CaHCT	ABO40491.1
Ipomoea batatas	IbHCT	BAJ14794.1
Leucaena leucocephala	LlHCT	AGA20364.1
Caragana korshinskii	CkHCT	AHK05938.1
Hibiscus cannabinus	HcHCT	AFN85668.1
Arabidopsis thaliana	AtHCT	At5g48930
Populus trichocarpa	PtHCT1	ACC63882.1
	PtHCT6	ACC63883.1
Arabidopsis thaliana	AtC3H1/AtCYP98A3	At2g40890
	AtC3H2/AtCYP98A8	At1g74540
	AtC3H3/AtCYP98A9	At1g74550
Salvia miltiorrhiza	SmCYP98A75	AJD25229.1
	SmCYP98A76	AJD25230.1
	SmCYP98A77	AJD25231.1
	SmCYP98A78/SmCYP98A14	ADP00279.1
Ocimum basilicum	ObC3H1	AAL99200.1
	ObC3H2	AAL99201.1
Phacelia campanularia	PcCYP98A111/PcC3H	QDF44409.1
	PcCYP98A112	QDF44410.1

	PcCYP98A113	QDF44411.1
Coffea Arabica	CaC3H	AFP49813.1
Sinopodophyllum hexandrum	ShCYP98A68	AGC29945.1
Populus trichocarpa	PtC3H3	ACC63870.1
Glycine max	GmCYP98A2	NP_001235563.1
Medicago truncatula	MtCYP98A37	ABC59086.1
Solanum lycopersicum	SlCYP98A2	XP_004230046.1
Coleus scutellarioides/Coleus blumei	CsCYP98A14/CbCYP98A14	CAD20576.2
Pinus taeda	PtaC3H	AAL47685.1
Selaginella moellendorffii	SmoC3H	EFJ17850.1
Physcomitrium patens	PpC3H	XP_024360823.1
Capsicum annuum	CaCYP98A2	PHT63449.1
Solanum tuberosum	StCYP98A2	XP_006364777.1
Sesamum indicum	SiC3H	AAL47545.1

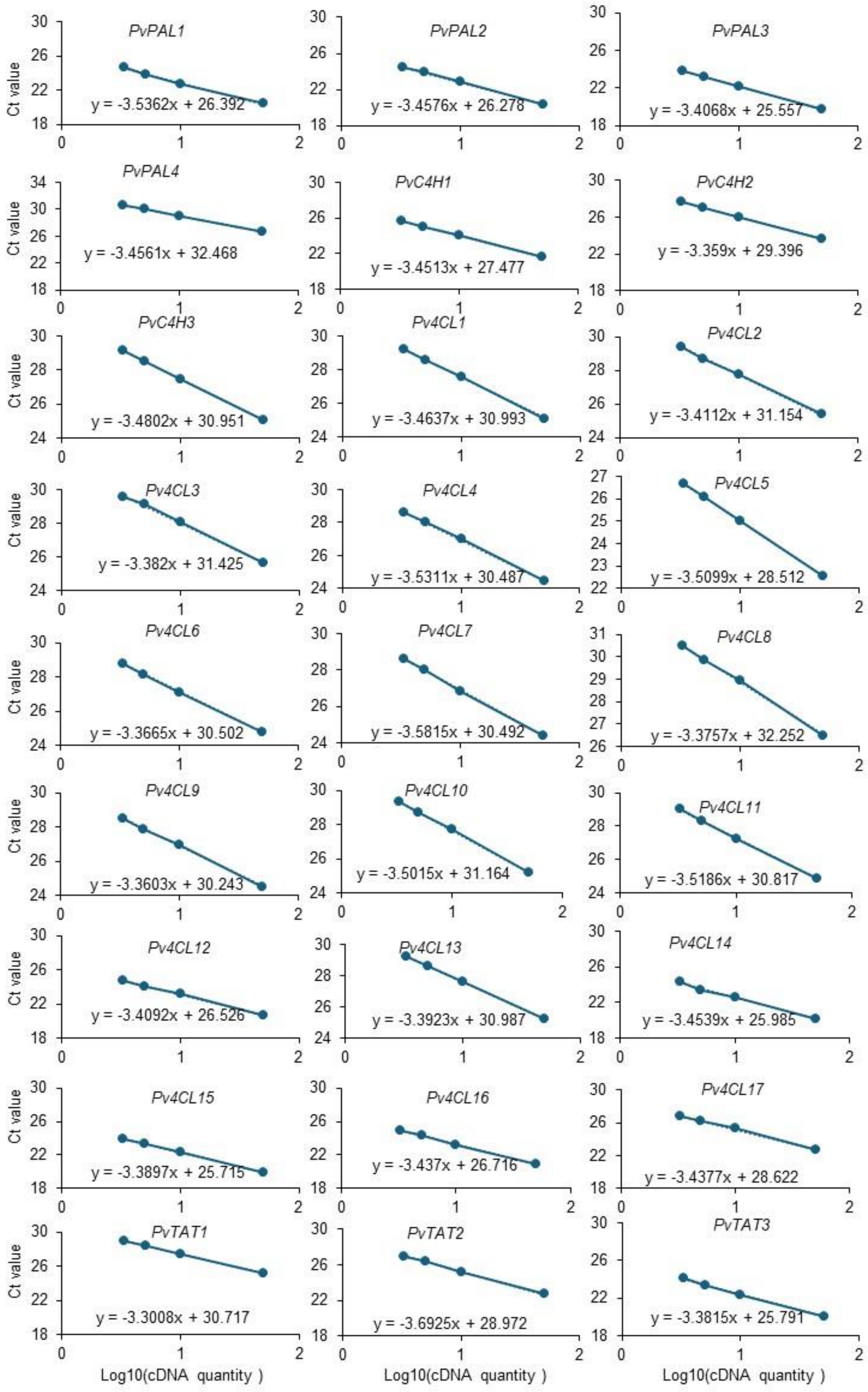
PvPAL1
y = -3.5362x + 26.392
PvPAL2
y = -3.4576x + 26.278
PvPAL3
y = -3.4068x + 25.557
PvPAL4
y = -3.4561x + 32.468
PvC4H1
y = -3.4513x + 27.477
PvC4H2
y = -3.359x + 29.396
PvC4H3
y = -3.4802x + 30.951
Pv4CL1
y = -3.4637x + 30.993
Pv4CL2
y = -3.4112x + 31.154
Pv4CL3
y = -3.382x + 31.425
Pv4CL4
y = -3.5311x + 30.487
Pv4CL5
y = -3.5099x + 28.512
Pv4CL6
y = -3.3665x + 30.502
Pv4CL7
y = -3.5815x + 30.492
Pv4CL8
y = -3.3757x + 32.252
Pv4CL9
y = -3.3603x + 30.243
Pv4CL10
y = -3.5015x + 31.164
Pv4CL11
y = -3.5186x + 30.817
Pv4CL12
y = -3.4092x + 26.526
Pv4CL13
y = -3.3923x + 30.987
Pv4CL14
y = -3.4539x + 25.985
Pv4CL15
y = -3.3897x + 25.715
Pv4CL16
y = -3.437x + 26.716
Pv4CL17
y = -3.4377x + 28.622
PvTAT1
y = -3.3008x + 30.717
PvTAT2
y = -3.6925x + 28.972
PvTAT3
y = -3.3815x + 25.791
Ct value
Log10(cDNA quantity)

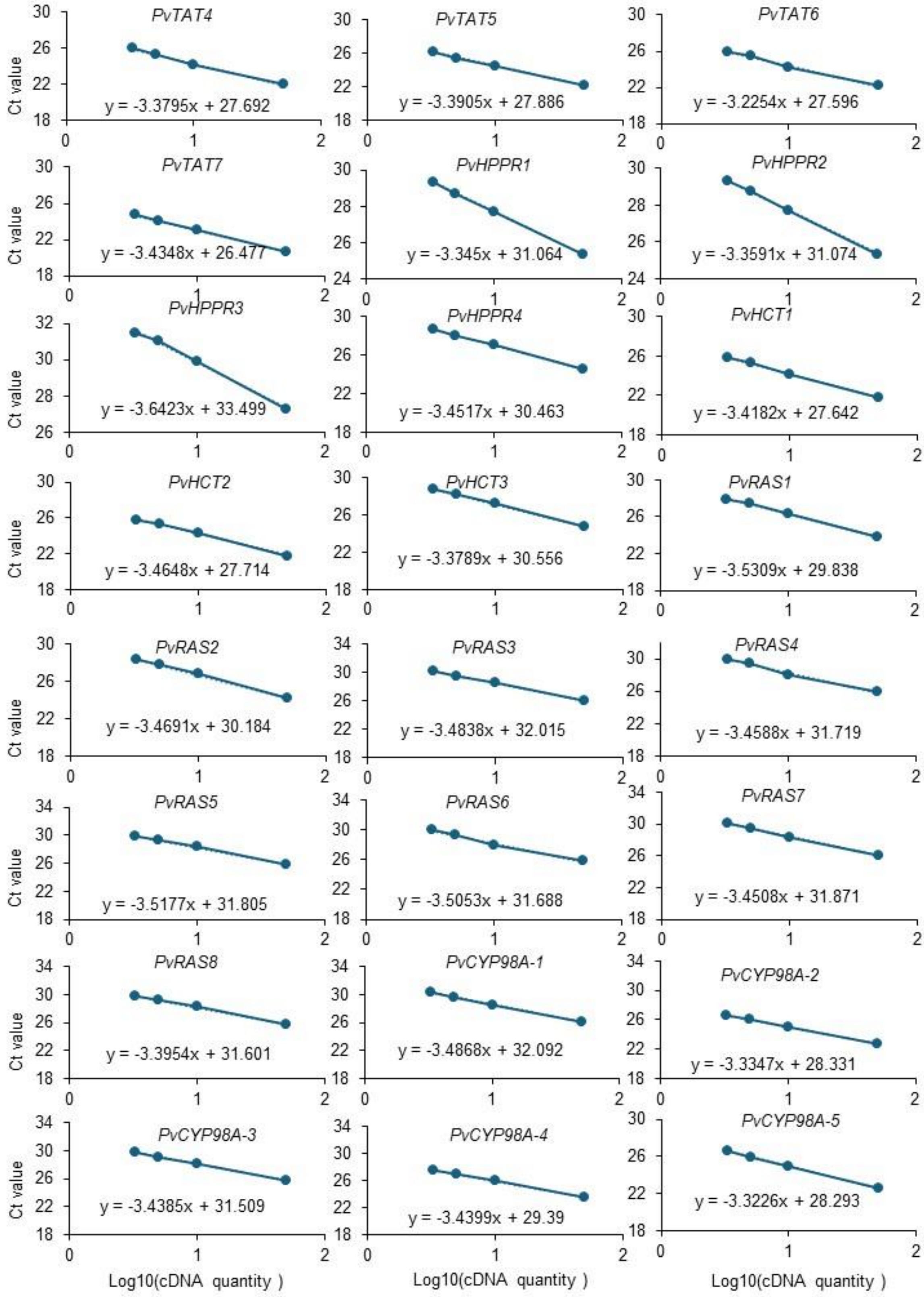

Fig. S1. Standard curves for evaluating the amplification efficiency of primer pairs on 51 RA biosynthesis-related genes. The Ct values were plotted against log of four different amounts of cDNA (50, 10, 5, and 3.3 ng).

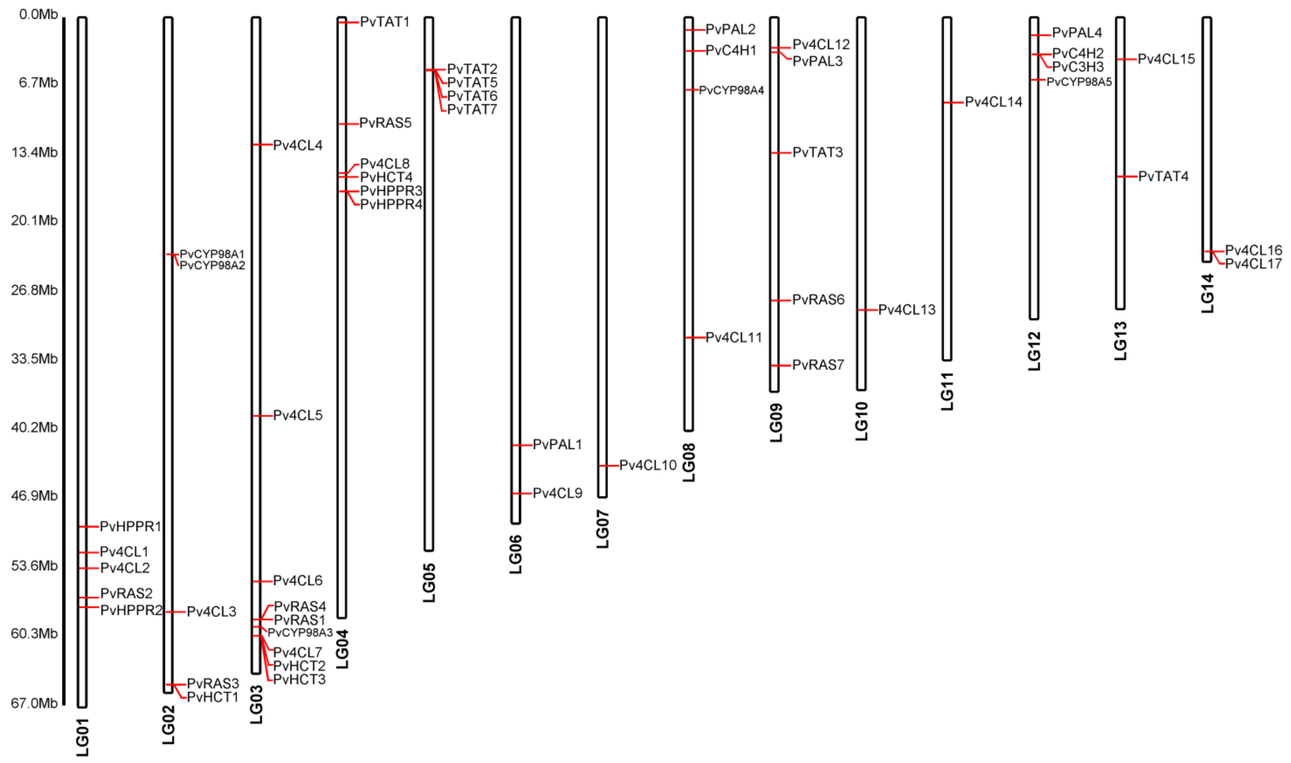

Fig. S2. Distribution of RA biosynthesis-related genes on the 14 chromosomes of *P. vulgaris*.

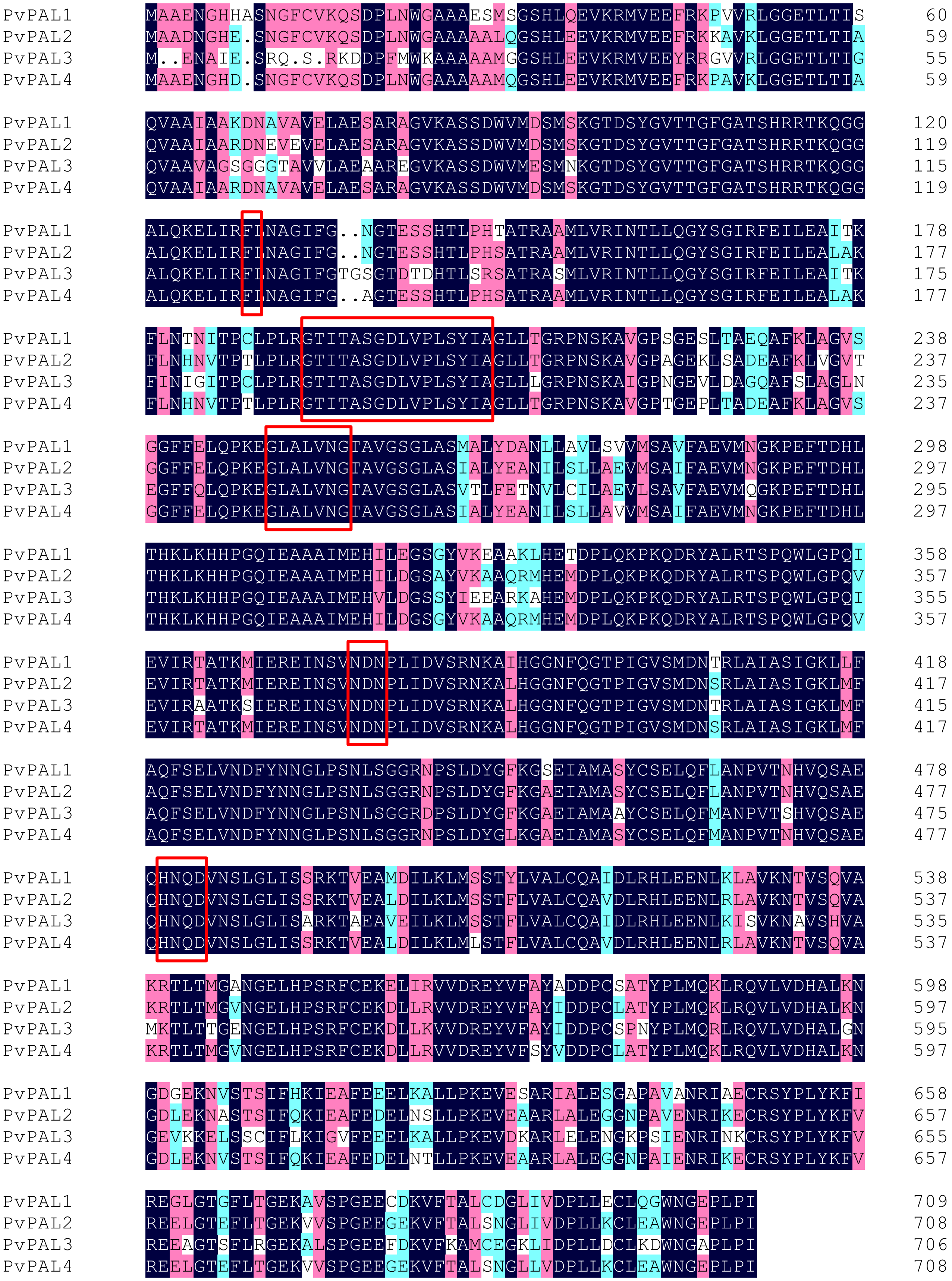

Fig. S3. Sequence alignment of four PvPAL proteins. The conserved motifs, including “FL”, “GTITASGDLVPLSYIA”, “GLALVNG”, “NDN” and “HNQD”, are shown in red boxes.

```
PvC4H1        MDLLLVEKTLIGLFFAIVVAAVVSKLRGKKFKLPPGPIPVPIFGNWLQVGDDLNHRNLTD     60
PvC4H2        MDLLLVEKTLVGLFFAIVVAAVVSKLRGKKFKLPPGPIPVPIFGNWLQVGDDLNHRNLTD     60
PvC4H3        MDFILFEKLLLSLLAAAVFAVAISKLRRQKLNLPPGPFPLPIFGSWLKVGNTFDQHDLAG     60

PvC4H1        YAKRFGDILLLRMGQRNLVVVSSPDLAKEVLHTQGVEFGSRTRNVVFDIFTGKGQDMVFT    120
PvC4H2        YAKRFGEILLLRMGQRNLVVVSSPDLAKEVLHTQGVEFGSRTRNVVFDIFTGKGQDMVFT    120
PvC4H3        MARKFGDVFLLRMGSRAITVASSPEAAKEVLLTQGKEFGSRSRNIVFDVFTGKGQDMIFT    120

PvC4H1        VYGEHWRKMRRIMTVPFFTNKVVQQYRHGWEAEAAAVIDDVKKNPESATNGIVLRRRLQL    180
PvC4H2        VYGEHWRKMRRIMTVPFFTNKVVQQYRHGWEAEAASVVDDVKKNPESATNGIVLRRRLQL    180
PvC4H3        EYGDHWRKMRRIVAVPFFTAKAVQHYRGDWEAEAAAVVAEMRGNAAAASEGVVLRRRLEL    180

PvC4H1        MMYNNMYRIMFDRRFESEEDPLFVKLKALNGERSRLAQSFEYNYGDFIPILRPFLRGYLK    240
PvC4H2        MMYNNMYRIMFDRRFESEDDPLFVKLKALNGERSRLAQSFEYNYGDFIPILRPFLRGYLK    240
PvC4H3        MIYNNVYKIMFDRKFESEEDPCYVKLKSVNAERSRLGQNLKYNFGDFIPILRPFLRGYLN    240

PvC4H1        MCQQVKERRLKLFKDYFVDERKKLVSTKPADKDGLKCAIDHMIEAQQKGEINEDNVLYIV    300
PvC4H2        MCQQVKERRLKLFKDYFVDERKKLVSTKPADKDGLKCAIDHMIEAQQKGEINEDNVLYIV    300
PvC4H3        ICREVTARRLNLYKDTFLQHRKNIASTKGIVNNGLKCGVDHFLEAQQNGEINEANVLYIV    300

PvC4H1        ENINVAAIETTLWSIEWGIAELVNHPEIQNKLRHELDTVLGPGVQITEPDIYKLPYLQAV    360
PvC4H2        ENINVAAIETTLWSIEWGIAELVNHPEIQNKLRHELDTVLGPGVQITEPDTHKLPYLQAV    360
PvC4H3        ENMNVAAIETTVWALEWAIAELINNPRVQEKLRAEIDTVLGSG.QVRETDTNKLPYLQAV    359

PvC4H1        VKETLRLRMAIPLLVPHMNLHDAKLGGFDIPAESKILVNAWWLANNPAQWKKPEEFRPER    420
PvC4H2        VKETLRLRMAIPLLVPHMNLHDAKLGGFDIPAESKILVNAWWLANNPAQWKKPEEFRPER    420
PvC4H3        IKETLRLRMVVPCLLPHMNLHQAKLGGYDIPSESRVLVNAWWLANDPTRWEKPEEFRPER    419

PvC4H1        FLEEEAKVEANGNDFRYLPFGVGRRSCPGIILALPILGITLGRLVQNFELLPPPGQKKID    480
PvC4H2        FLEEEAKVEANGNDFRYLPFGVGRRSCPGIILALPILGVTLGRLVQNFELLPPPGQSKID    480
PvC4H3        FLEEESKVEATGNDLKYIPFGVGRRSCPGIVIAMPVLGITLGRLVQNFELLPPPGQSKID    479

PvC4H1        TSEKGGQFSLHILKHSTIVMKPIS                                        504
PvC4H2        TSEKGGQFSLHILKHSTIVMKPIS                                        504
PvC4H3        TTENNGQFATRILKHYSVVMKAIN                                        503
```

Fig. S4. Sequence alignment of three PvC4H proteins. The five conserved P450 motifs, including the proline-rich motif "PPGP", the oxygen binding motif "AAIETT", the "ETLR" motif, the "PERF" motif and the heme-binding motif "FGVGRRSCPG", are shown in red boxes.

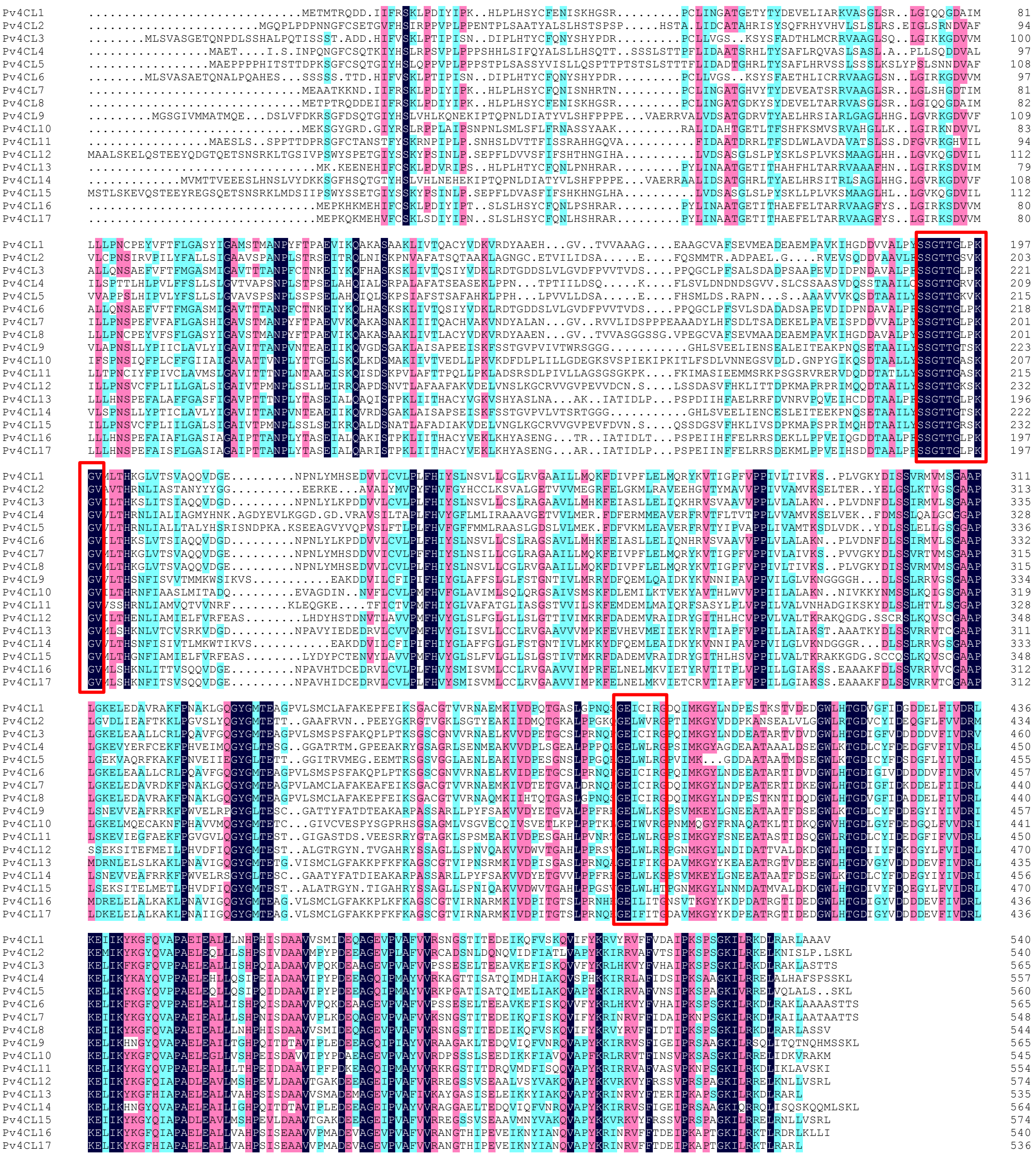

Fig. S5. Sequence alignment of seventeen Pv4CL proteins. Two conserved motifs, including Box I with the representative sequence “SSGTTGLPKGV” and Box II with the representative sequence “GEICIRG”, are shown in red boxes.

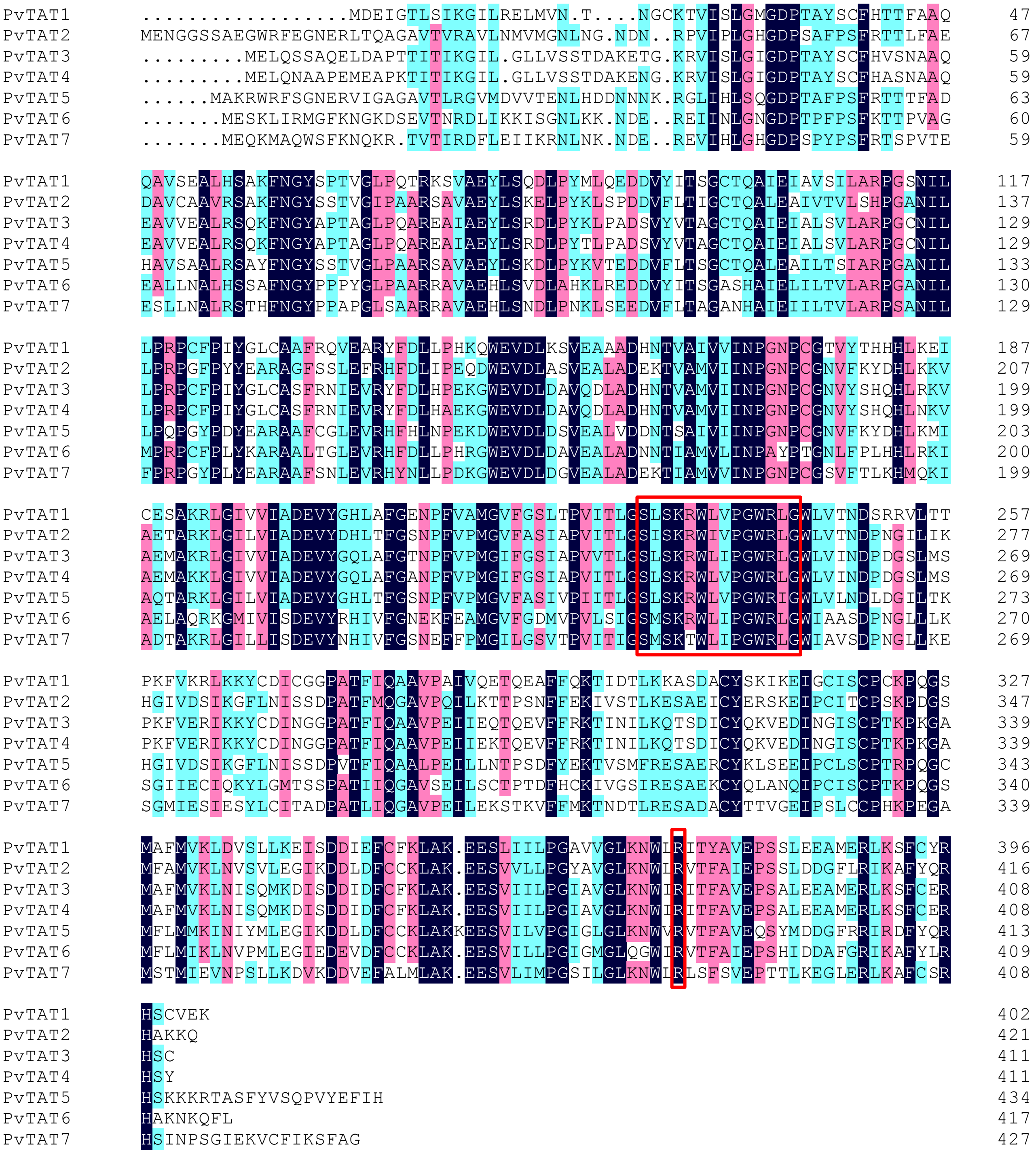

Fig. S6. Sequence alignment of seven PvTAT proteins. Two conserved motifs, including Motif I with the representative sequence “SLSKRWLVPGWRLG” and Motif II with the Arg residue, are shown in red boxes.

```
PvHPPR1   MD..A..FGVLMMYPMSNYLEPELDSRFKLFRYWPHPNQ.P.EFLAEHAASIRAVVGNAS    54
PvHPPR2   ME..S..VGVMMTTPMSAYLEHQLQQRFILFKLWESPSH.S.QFLTRCRDSIKAVVGDTR    54
PvHPPR3   MD..A..IGVLMMCPMSNYLEQELDNRFKLFRYWAQPKQ.R.DFLADHAASIRAVVGNAT    54
PvHPPR4   METISNPIGVLLLRPLSPYIQQQLSSRYALFRFWESPPHLRLDFLRQHSASIKAVVPNGV    60

PvHPPR1   A....ELIDALPELEIVSCFGVGFDKVDLIKCKERGIRVTNTPDVLTDDVADLAIGLMLA   110
PvHPPR2   IGADSELIDSLPRLEIVATYSVGLDKIDLGKCGERGIRVTNTPDVLTDDVADLAIGLALA   114
PvHPPR3   AGADAELIASLPKLEIVSCFSVGLDKVDLIKCKEKGIRVTNTPDVLTDDVADLAIGLILA   114
PvHPPR4   QGVDAEIIDALPLLEIIASHSSGLDKIDLDRCRRRGIKVTYTPDALTDEVADMAILLILA   120

PvHPPR1   VLRRICECDKYVRRGAWKLGDFKLTTKFSRKRVGIIGLGRIGLAIAERAEAFDCPISYHS   170
PvHPPR2   TLRRICACDAFVRSGSWRNGDFLLTTKFSGKSIGILGLGRIGSAIARRAKAFGCTIGYHS   174
PvHPPR3   VLRRICECDKYVRRGAWKLGDFKLTTKFSGKRVGIIGLGRIGLAVAERAEAFDCPISYYS   174
PvHPPR4   TCRRICAADNFVRRGDWKNSDFLLTTKFSGKSVGIIGLGRIGSAIAKRVEAFGCRIGYHS   180

PvHPPR1   RSKKGNPNYTYYDSVVELASNSDILVVACALTPETTHIVNRQVIDMLGPKGILINVGRGV   230
PvHPPR2   RTKKQNTSYTYFSNTVDLAANCQILFVTCALTDETRHIVNREVIDALGASGVLVNVGRGA   234
PvHPPR3   RSKKGNTNYTYYGSVVELASNSDILVVACALTPETTHIVNRQVIDALGPKGILINIGRGP   234
PvHPPR4   RSPKPNSRYKYYSSVIDLALHSQILVVSCALTPETHHIVDRGVMDALGRDGIIVNIARGS   240

PvHPPR1   HVDEAELVSALVEGRLGGAGLDVFEKEPELPEQLFELENVVLLPHVGSGTEETRKAMADL   290
PvHPPR2   LVVEHELVSALLEGRLGGAGLDVFENEPHVPESFFGLHNVVLLPHVGTDTVETSDAMADL   294
PvHPPR3   HIDEAEMVSALVEGRLGGAGLDVFEKEPEVPEQLFGLENVVLLPHVGSGTEETRKAMADL   294
PvHPPR4   HIDQAELIRALAEGRLRGAGLDVLEHEPQVPDLLAGLDNVVLSPHVGATTFETRKRLADL   300

PvHPPR1   VLGNLEAHFSSKPLLTPV                                             308
PvHPPR2   VVQNLECHFLKKPLLTPV                                             312
PvHPPR3   VLGNLEAHFSSKPLLTPV                                             312
PvHPPR4   VIANLEAHFANEPLLNQV                                             318
```

Fig. S7. Sequence alignment of four PvHPPR proteins. The NAD(P)H binding motif with the representative sequence "GLGRIG" and the putative myristylation site with the representative sequences "GTVETR" and "GNLEA" are shown in red boxes.

```
PvHCT1    MKIEVRDSTLVRPAAATPAVSLWNSNVDLVVP.NFHTPSVYFYRPNGE...ASFFNTAVMKAALGRALVPFYPMAGRLKR    76
PvHCT2    MKIEVRESTLVRPSEATPAVSLWNSNVDLVVP.NFHTPSVYFYRPNGE...ADFFDTAVMKAALGRSLVPFYPMAGRLKR    76
PvHCT3    MKIYVKESTLVTPASETPSLSLACSNLDLVMS.CSHMASVYFYRPNGGG..DHFFNTAVLKVALSHTLVPFYPLAGRLRV    77
PvRAS1    MSLKVKESTMVKPTAETPSGSVWLSSLDLLMPATHHTRSIYFYRPDAA.AAANFFDAEVLKAALGRALVDFYPYAGRLNK    79
PvRAS2    MKISVKASTIVKAMKESHTESVWLSSLDLLLPDSAYHSRSVYFYRNSA.AAANFFDAEVLKAALSQNLSEFYPFAGRLRK    79
PvRAS3    MKIEVKESTMVKPAAETPTGGLWISNLDLLSPANYHTLSVHFYRHDG...SDNFFEAAPLKEALSRALVEFYPYAGRLKL    77
PvRAS4    MKITVKETTMVKPMEETPGGTLWLSNLDLQMPTTYHSRSVYFFPHDS...SADFFDAAVLKAALSRTLVLFYPFAGRLRQ    77
PvRAS5    MKIIVKESKMVKPMEETPSGSVWLSNLDLVIP.SNHHALCLYFYR.CN.GAADFFDTTVFKAIMSRALVYFYPYAGRLRK    77
PvRAS6    MKIIVKETTMIKPAEETPSGTVWLSNLDLLMPATYHSRSVYFYPHDG...SANFFDVMVLKAALSCALVRFYPLAGRLGR    77
PvRAS7    MKITVKETTMVKPAEETPSGTVWLSNLDLLMPATYHSRSVYFYPHDG...SANFFDVTVLKAALSRVLVRFYPLAGRLGR    77
PvRAS8    MKIEVKETTVVKPSPENPRNRMWISNADDDFLENFHMRTHTFYRPAA....ADFFNTAVLKAALARTLSDFYPIAGRLSK    76

PvHCT1    DEDGRIEIDCNAEGVLFVEAVSDGSVDDYGDF.APNLELRRLIPAVDYSQGISAYALLVLQVTFFKCGGVSLGVGMQHHV   155
PvHCT2    DEDGRIEIDCNAEGVLFVEAVSDGSVDDYGDF.APNLELRRLIPAVDYSKGISAYPLLVLQVTFFKCGGVSLGVGMQHHV   155
PvHCT3    AGDGRLKIDCNAKGVLFVAAECDAVLDDLTDF.APRMELRRLVPAVDYSRGVSEYPLSVVQVTHFKCGGVCLGAGNHHNV   156
PvRAS1    ADDCRIQINCNSEGVLFVEADCDAAIDDLPAG.FQPTPDLLLVPKVDYSLGISSFPLLLLQLTRFKCGGACLGVANEHHI   158
PvRAS2    DDNGRLEINCNNEGALFIEAECDGTIDDFG...FTPRPDLSLLPLIDYSPGISTFPLCLVQLTRFKCGGVSLSFSNEHHV   156
PvRAS3    NGK.RLEIDCNGEGLLLVEAESDGTLEELGD..FAPRPDLNLIPKVDYSKGISSYPLMLFQITRFKCGGVGLGVANEHHL   154
PvRAS4    DNEGRLQIHCNAQGAVFVEAECDATLADLGD..FSPTPDITFAAKADYTQGLYNWPLSLLQLTRFKCGGVSLGVTNDHHV   155
PvRAS5    DNEGRLEINCNGEGVLFVEAECDGALDDLGG..FSPRSDISLLPSVDYSKGLSTYPLLLVQLTRFKCGGVSLGFANEHHV   155
PvRAS6    DGEGRLEINCNNEGVVFVEAECDATLEDLGD..FSPRAEISFAPKVDYSRGLSTWPLSLLQLTRFKCGGVSLGVTNDHHL   155
PvRAS7    DGEGRLEINCNNEGVVFVEADCDATLEDLGD..FSPRVEISFVPKVDYSQGLFTWPLSLLQLTRFKCGGVSLGVTNDHHL   155
PvRAS8    DDNGRVEIDCNGEGAVFLEAEADGAIDDLGDFRPDPRPDSGLAPQVDYSQALSLFPLLLVQVTRFKCGGVCFGVTMAHKV   156

PvHCT1    ADGFSGLHFINAWSDMARGLDIT..VPPFIDRT.LLRARDPPQPEFKHVEYQPPPS....MKTYGAAETVVSIFRLTREE   228
PvHCT2    ADGFSGLHFINTWSDMARGLDIT..LPPFIDRT.LLRARDPPQPQFKHVEYQPPPS....MKTYTTAETVVSIFKLTRDL   228
PvHCT3    VDGYSGLHFINSWSRVSRGLDIA..VPPFIDRA.LLNPRRPPCPNFKHIEYEYKP.....NPFSRQTETTVSMFELTRDQ   228
PvRAS1    SDGISALHFINTWSHIARHGLTSSTHPPFLDRR.VLSPRLPPHHHFPHIEYQPPPPPTTPLPTSHG..ATHSTFTLTPHH   235
PvRAS2    ADGASALNYINTWSDIAYGRATSAAVPPFLDRR.LLTARNPPQPRFPHIESQPPPPLKTPLPDSEAAATSSKTFRLTRDQ   235
PvRAS3    SDGVSALHFINTWCHLARGVPAPS.PAPVFDRT.ALSARSPPKVEFTHPEFQPPPTLPTPLEHTD...IAYAKLKLSRDQ   229
PvRAS4    CDGASGLNYVNTWSQIARGITTP..PPPFIDRR.ILAARTPPQPKFTHTEYLPPP..SVTIDEATP..SAFKTFTLTLEN   228
PvRAS5    ADGSSGLLFAKTCSDIARG..GVPTVPPFLDRRSLLSAREPPRPMFPHIEHHPPPSLKTPLTNS...DTTFSTFKLTRDQ   230
PvRAS6    SDGKATLDYINTWSDIAAGVTPLR.PPPYMDRR.VLAARFPPQPKFSHIEYLPPPS.LGTPLDIEE..KAFKTFTLTRTH   230
PvRAS7    SDGTAALDYINTWSDIAAGVTPLR.PPPYMDRR.VLAARFPPQPKFSHIEYLPPPS.LGTPPDIEE..KAFKTFSLTRTH   230
PvRAS8    QDGISAYHLLTTWADIARGGGGD..MKPYLDRR.VLAARNPPQPKFDHLEFQPPP..LLKHSNYNVSETKFSMLKLSREQ   231

PvHCT1    LTTLKAKSKD.........EGNTVTYSSYEMLAGHVWRCACLARGLPEDQETKLYIATDGRSRLQPPLPPGYFGNVIFTA   299
PvHCT2    LTTLKAKSKD.........EGNTVTYSSYEMLAGHVWRCACLARGLPEDQETKLYIATDGRSRLQPPLPPGYFGNVIFTA   299
PvHCT3    LNTLKSKCTG.........AGDKAAPTSYELLAAHIWRCVCRARLLREGEETRLYIAADGRTRLQPPLPQGYFGNVIFTA   299
PvRAS1    LNSLKQKCN...........QNNINYTTYEIVAGHLWRCVCMARDLPHDQQTKLQIPVDGRRRLRPPLPPGFFGNGIFYA   304
PvRAS2    INALKHKCNSNKDD...DHTNKLINYTSYEAIAGHVWRCATISRKLPADQETKLQLPVDGRQRLKPPLPRGYFGNVAFYT   312
PvRAS3    LNALKAKCKSPATANGQENGKPARPYSTFEVLAGHIWRCVCAARGLPAEQETKLHIPFDG..RAKLQLPPGFFGNAIFFA   307
PvRAS4    LKDIKQSCN...........SGGASYTTYEAVAGHLWRCICMARRLPHEQETKMQIPVDARTRLKPKLPRNYFGNAVFYA   297
PvRAS5    INALKQNCSN..........SHRSNYTSYEVVAGHVWRCVTIARGLPGDQETKLQLLVDGWQRLRPPLPLGFLGNGIFYT   300
PvRAS6    IEEIKRKCKN.........SSGGVRFTTYEAVAGHLWRCICKARQLPHDQETKIQIPVDARARLRPALPPHYFGNGVFYA   301
PvRAS7    IEEIKRKCNN.........SSGGVRFTTYEAVAGHLWRCICKARQLPHDQETKMQIPVDARARLRPALPQHYFGNGVFYT   301
PvRAS8    LNTLKHKCKEQDQE...DDANMPLSYTSFETITGHAWKCICKARGLEKDEESRLIIMVNGRTRLRPPLPPGYYGNVIFKA   308

PvHCT1    TPLAVAGDLESKPVWYGASKIHDALARMDNEYLRSALDYLEMQPDL.....KALVRGAHTFRCPNLGITSWVRLPIHDAD   374
PvHCT2    TPLAVAGDLESKPVWYGASKIHDALARMDNEYLRSALDYLELQPDL.....KGLVRGAHTFRCPNLGITSWVRLPIHDAD   374
PvHCT3    MRAGVSHDLVSEPLWRVAGEIHEALVRMDDEYLRSALDFLEVQADL.....KALVRCEGTYRNPNLGITSWARLPVYEAD   374
PvRAS1    TSAAFCGELVSNPLEYSVAKVHEALARMDDDYLRSAIDYLEARVPD....ISDIARSENNVRCPNFGITSWVRLPFYEAD   380
PvRAS2    ATIALCGKLESNPLRFAAGKVHEAIAQMDDDYLRSALDYSELRLSN...VDSAAARVENRVKCPNLAITSWGRLPFYEAD   389
PvRAS3    TPIATCGEVEANSLSYAVRRVGDAIGRLDEEYLRSSVDFLELQDDI.....SKLAQGAHSFRCPNLWVISWVRLPIYEPD   382
PvRAS4    TPVALCGEIETKPLEFAVGKIHEAVARMDDEYMRSALDYVELRLSD....ISAIARSEANVRCPNVGITSWTRLPFYEAD   373
PvRAS5    AAFAVCGELGSSPLRFAVGKVQEAIARMNNEYLRSSLDYLELQLHNNRETDEGIVRSERNVKCPNLAITSWVRLPLYGID   380
PvRAS6    TPLALCGEIESNPLRFAAGKIHEALVRMDDEYMRSALDYVEAQVSN....ISAIARSEANVRCPNAGITSWTRLPFYEAN   377
PvRAS7    TALALCSEIESNPLRFAAGKIQEALARMDDEYMRSALDYVEARVSN....ISAIARSEANVRCPNAEITSWTRLPFYEAD   377
PvRAS8    AGVASCGEIESNPLKFAVGKVREMVGRMDDEYLRSAIDYLEVRGGVG.....PEMRGTIIHKSPNMGITSWARLPLYDID   383

PvHCT1    FGWGRPIFMGPGGIAYEGLSFVLPSPANDGSLSVAISLQAEHMKLFEKLLYEI     427
PvHCT2    FGWGRPIFMGPGGIAYEGLSFVLPSPSNDGSLSVAISLQAEHMKLFQKLLYEI     427
PvHCT3    FGWGKPAFMGPAGIPFEGLCYVLGNPANEGGVAVAIRLEVEHMELFGKLLYDI     427
PvRAS1    FGWGKPVYAGPGAAQFEGKALLFVDKESEGRLLLAITLLKPHMEAFQHLLYHV     433
PvRAS2    FGWGKPVYAGPGVALFEGKSFLFSDHENEANMMLAITLHKKHMDLFEKILYDI     442
PvRAS3    FGWGKAVYMGPWAAPFEGKSYLLPNPDNDGSLFVAITLHTQHMDRFQNFFYDI     435
PvRAS4    FGWGKPDFAGPAAAPFEGRGYLAIDPKTEGHMLLTIALLKPHLDLFQKLLYDI     426
PvRAS5    FGWGKPVYAGPGAPLIEGKSYLLADIEDEGSMLVAITLHKHHMEPFQKLLYNVH   434
PvRAS6    FGWGKPVFAGPAVAPFEGRGYLAMDPKNEGEMLLTIALLKPHLEVFENFLYHII    431
PvRAS7    FGWGKPVFAGPAVAPYEGRGYLGMDPKNEGGMLLTIALLKPHLEVFENILYHII    431
PvRAS8    FGWGKPFYVGLAFVPVDGHLILLPSPVTDGSLSLAIALPEDQMKLFHNCFYHI     436
```

Fig. S8. Sequence alignment of four PvHCT and seven PvRAS proteins. The conserved “HXXXD” and “DFGWG” motifs are shown in red boxes.

```
PvCYP98A-1   MAALLLLLLSLPL.AAVLLYHLLFRLRFRIPPGPFPWPVVGNLYHIKPVRFRCFAEWAQS    59
PvCYP98A-2   MAPLSLFLLSIPL..LFLIHSLYYRLRFRIPPGPRGWPVVGNLYDVKPLHFRCFAEWAQS    58
PvCYP98A-3   MGVLFLIVL.V.LAAVFAGYKVLKRLRYKIPPGPRPLPIVGNLYDIKPVLVRCFTEWAET    58
PvCYP98A-4   MAALLLLLL.LPLSALFLLHHLYYRLRFRIPPGPRPLPIVGNLYDIKPVRFRCYADWAQS    59
PvCYP98A-5   MAALLLLLL.LPLPALFLLHHLYYRLRFRIPPGPLPLPIVGNLYDIKPVRFRCYADWAQS    59

PvCYP98A-1   YGPIISVWFGSTLNVIVSNSELAKEVLKEKDGQLSDRHRSRSAVKLSRDGKDLIWADYGP   119
PvCYP98A-2   YGPIISVWLGSTLNIVVSSSDLAKEVLKEKDQQLADRYRSRTATRLSKYGQDLIWADYGP   118
PvCYP98A-3   YGPIFSVYLGSHLSVVVNSAGLAKEVLKDNDVQLAYRNRTRTINKFSKNGMDLIWADYGP   118
PvCYP98A-4   YGPIISVWFGSTLNVVVSNTALAKEVLKEKDQQLADRHRSRSAAKFSRDGQDLIWADYGP   119
PvCYP98A-5   YGPIISVWFGSTLNVVVSNTALAKEVLKEKDQQLADRHRSRSAAKFSRDGQDLIWADYGP   119

PvCYP98A-1   HYVKVRKVCTVVLFSPKRLELLRPIREDEITTMVESIYNDCTASGNSGKSVVIKKYLASM   179
PvCYP98A-2   HYVKVRKVCTVALFSAKSLESLRPIREDEVSAMVESIYNDYTNSGNSGKSLLLRKYLLAV   178
PvCYP98A-3   HYVKVRKLCTLELFSIKRLDALRPIREDEVTDMVHSIFQDSSKPENKGKAVVLREYLGMM   178
PvCYP98A-4   HYVKVRKVCTLELFSPKRLEALRPIREDEVSAMVESIYNDCTASDNLGKSLLVKKYLGAV   179
PvCYP98A-5   HYVKVRKVCTLELFSPKRLEALRPIREDEVSAMVETIYNDCTSSDNLGKSLLVKKYLGAV   179

PvCYP98A-1   AFHNITRLVFGKRFVDSDGA..VDKQGQEFKAIAINGLRLGASL.AMAEHIPWLRWAFPL   236
PvCYP98A-2   SFNNITRLAFGKRYVDTEGEGRVHKQGEEMRAIAENRLRIGASR.PIAEHVPWLRWMFPL   237
PvCYP98A-3   AFLHITRLTFGKRFIDSNGV..IDEQGQELKYILNNAITLGTKKSAFAEFLPWLNFLFKE   236
PvCYP98A-4   AFNNITRLAFGKRFVNSEGV..IDKQGLEFKAIVSNGLKLGASL.AMAEHIEWLRWMFPL   236
PvCYP98A-5   AFNNITRLAFGKRFMNSEGV..IDKQGLEFKAIVSNGLKLGASL.AMAEHIEWLRWMFPL   236

PvCYP98A-1   DEDAFTQHGVRMERLTRDIMKEHTLLRQKTGDAKQHFFDALLTLKDEYDLSEDTIIALLW   296
PvCYP98A-2   NEEDFVKHAARRDRLTREIMEDHNLARKKTGGAKQHFCDALLTLKDKYDLSDDTIIGLLW   297
PvCYP98A-3   QNEALAAHDARANSFTKRIMEEHTLARQKTGNTKNHFVDALLTLQDEYQLSDDTVTGLLW   296
PvCYP98A-4   DEDAFAKHGARRDTLTREIMEEHTLARQKSGGAKEHFFDALLSLKDKYDLSEDTIIGLLW   296
PvCYP98A-5   DEDAFAKHGARRDTLTREIMEEHTLARQKSGGAKEHFFDALLSLKDKYDLSEDTIIGLLW   296

PvCYP98A-1   DMIAAGMDTPAISVEWAMAELVRNPRVQQKVQEELDRVIGQDRVMTEVDIPNLPYLQCVV   356
PvCYP98A-2   DMIHAGMDTTAIIVEWAMAELIKNPRVLEKLQEELDSVIGTDRLMTELDVANLPYLRCVV   357
PvCYP98A-3   DMISAGMDTTTITAEWAVAEIVRNPRVQQKVQEELDRVVGRDRVMTEADIANMPYLQGVT   356
PvCYP98A-4   DMITAGMDTTAISVEWAMAELIKNPRVQQKAQEELDRVIGYERVITELDFSNLPYLQCIA   356
PvCYP98A-5   DMITAGMDTTAISVEWAMAELIKNPRVQQKAQEELDRAIGYERVITELDFSNLPYLQCIA   356

PvCYP98A-1   KEALRLHPPTPLMLPHRANSNVKIGGYDIPKGSNMNVNVWAVARDPAVWKNPLEFRPERF   416
PvCYP98A-2   KESLRLHPPTRLMLPHRANANVKVGGYDIPKGATVHVNVWAIARDPKVWKNPSEFRPERF   417
PvCYP98A-3   KECYRLHPPTPLMLPHKASTDVKIGGYDVPKGATVSVNVWAIARDPAVWKNPLEFRPERF   416
PvCYP98A-4   KEALRLHPPTPLMLPHRANTNVKIGGYDIPKGSNIHVNVWAVARDPAVWKNPSEFRPERF   416
PvCYP98A-5   KEALRLHPPTPLMLPHRANTNVKIGGYDIPKGSNIHVNVWAVARDPAVWKNPSEFRPERF   416

PvCYP98A-1   LEEDVDIKGHDFRLLPFGAGRRVCPGAQLGLDLTTSMIGHLLHHFSWGPPKGMRAEEIDL   476
PvCYP98A-2   LEDDVDIKGHDFRLLPFGAGRRICPGAQLGLDMVTSMVGHLVHHFKWAPPSGVSTESIDF   477
PvCYP98A-3   LEEDVDMRGTDYRFLPFGSGRRICPGAQLAINLVTSVLGHMLHHFTWSPSSGEKPEDIDM   476
PvCYP98A-4   LEEDVDMKGHDFRLLPFGAGRRVCPGAQLGINLVTSMIGHLLHHFNWAPPNGMSTEEINM   476
PvCYP98A-5   LEEDVDMKGHDFRLLPFGAGRRVCPGAQLGINLVTSMIGHLLHHFNWDPPKGMRTEDIDM   476

PvCYP98A-1   AENPGTVTYMKNPVEALPVPRLAPHLYKRVAV.D                             509
PvCYP98A-2   TERPGVVTFMKTPLETVANPRLPANLYRRIAV.D                             510
PvCYP98A-3   MEQPGTVTYMRKPLEAIPTPRLPVELYKRVAAGN                             510
PvCYP98A-4   GENPGLVTYMQTPLEAVPTPRLPAVLYKRIAT.D                             509
PvCYP98A-5   GENPGLVTYMRTPLEAVLTPRLPAVLYKRITA.D                             509
```

Fig. S9. Sequence alignment of five PvCYP98A proteins. Five conserved P450 motifs are shown in red boxes.

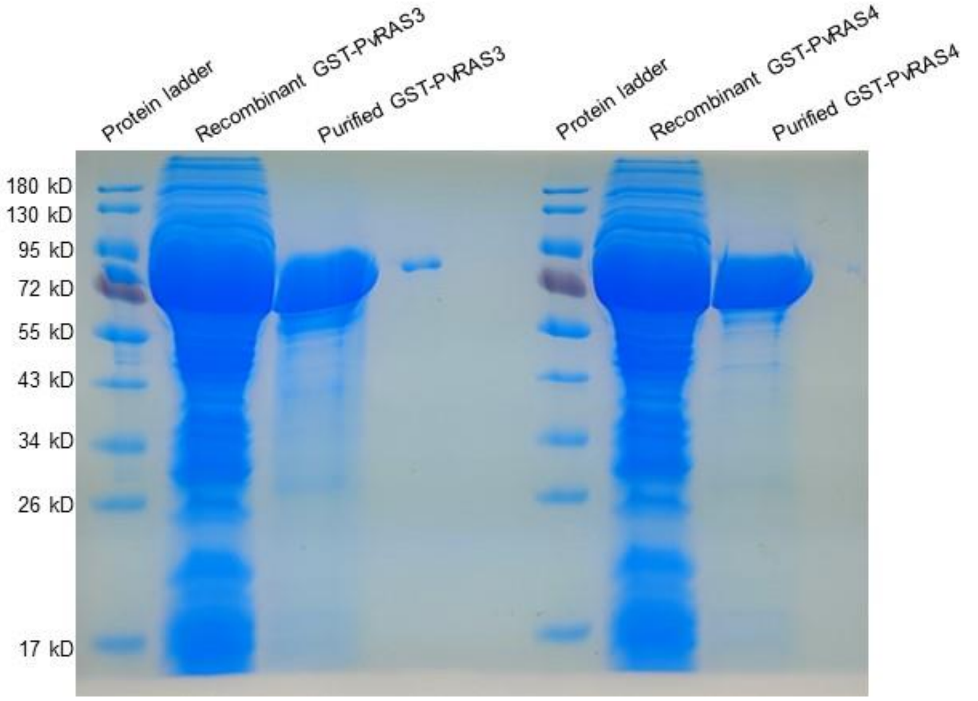

Fig. S10. Analysis of purified recombinant PvRAS3 and PvRAS4 proteins on a Bis-Tris Plus Gel.

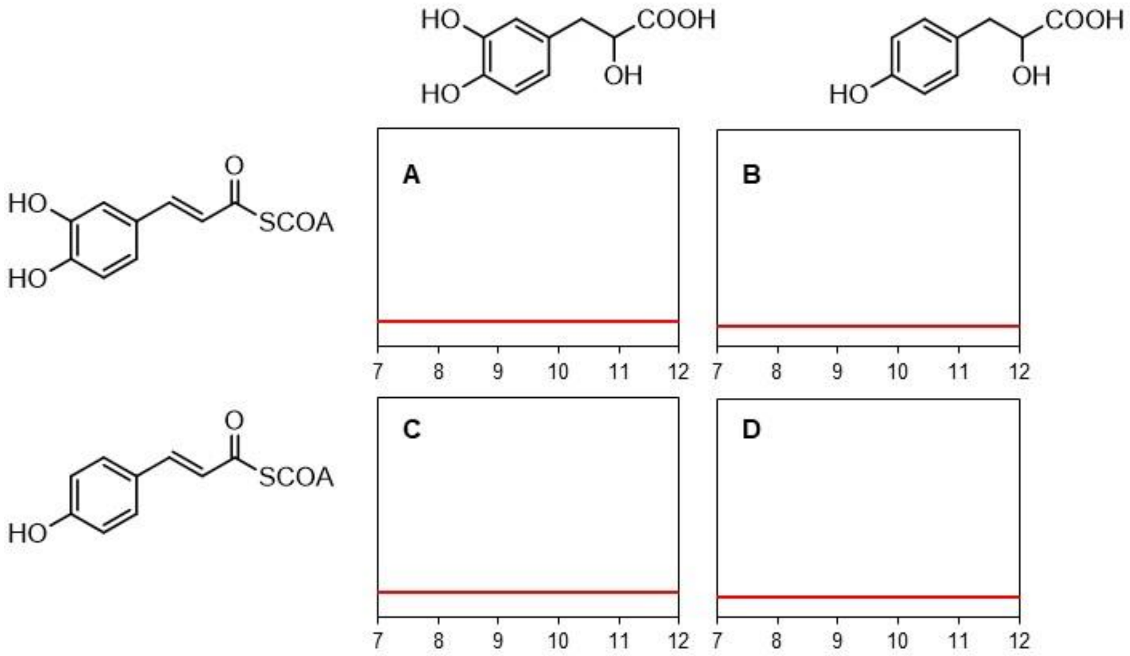

Fig. S11. UPLC chromatograms of the reaction products with total proteins from *E. coli* transformed with the empty pGEX-4T-1 vector.

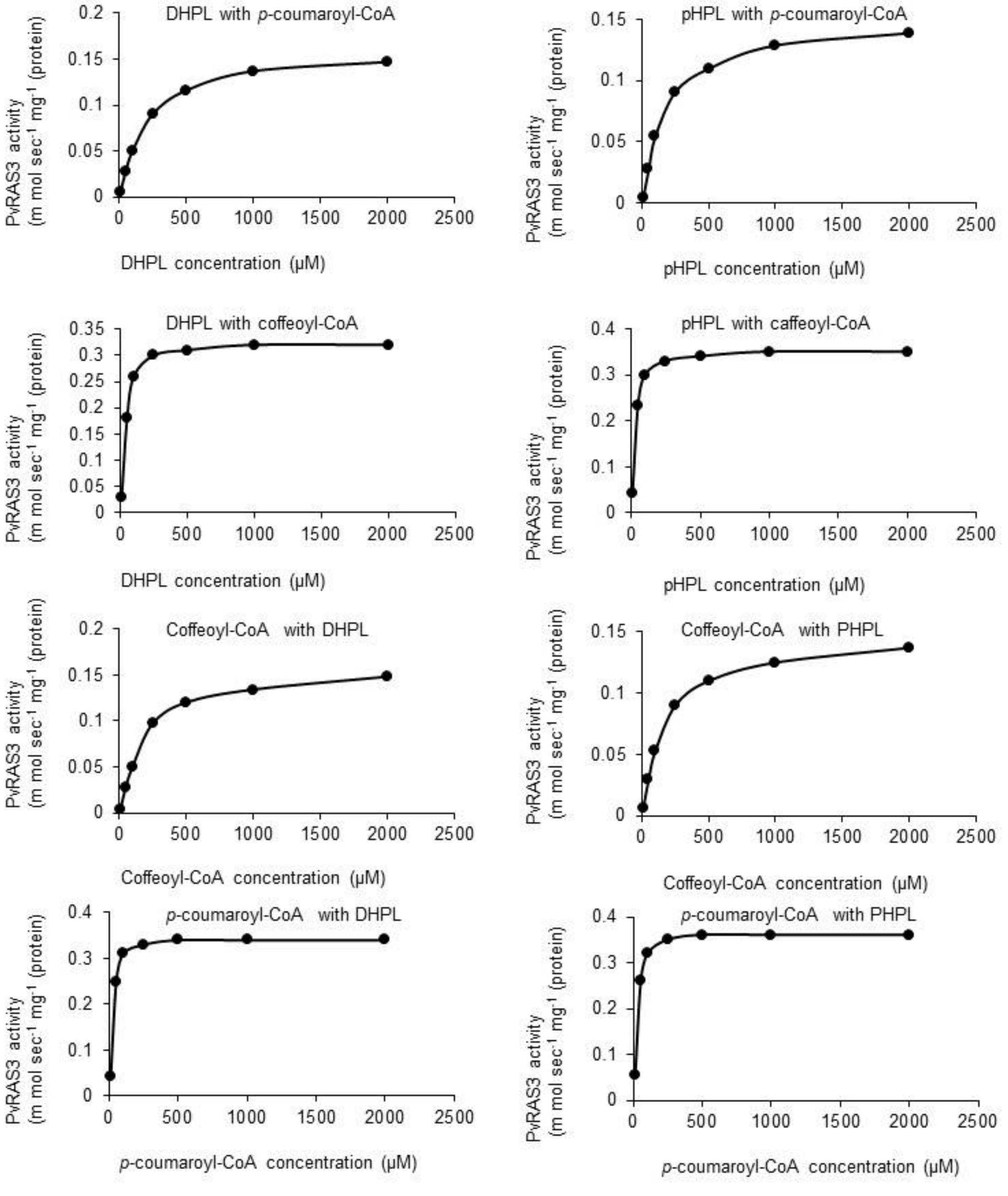

Fig. S12. Plots of initial velocity versus substrate concentration for recombinant PvRAS3 with donors and acceptors as substrates.

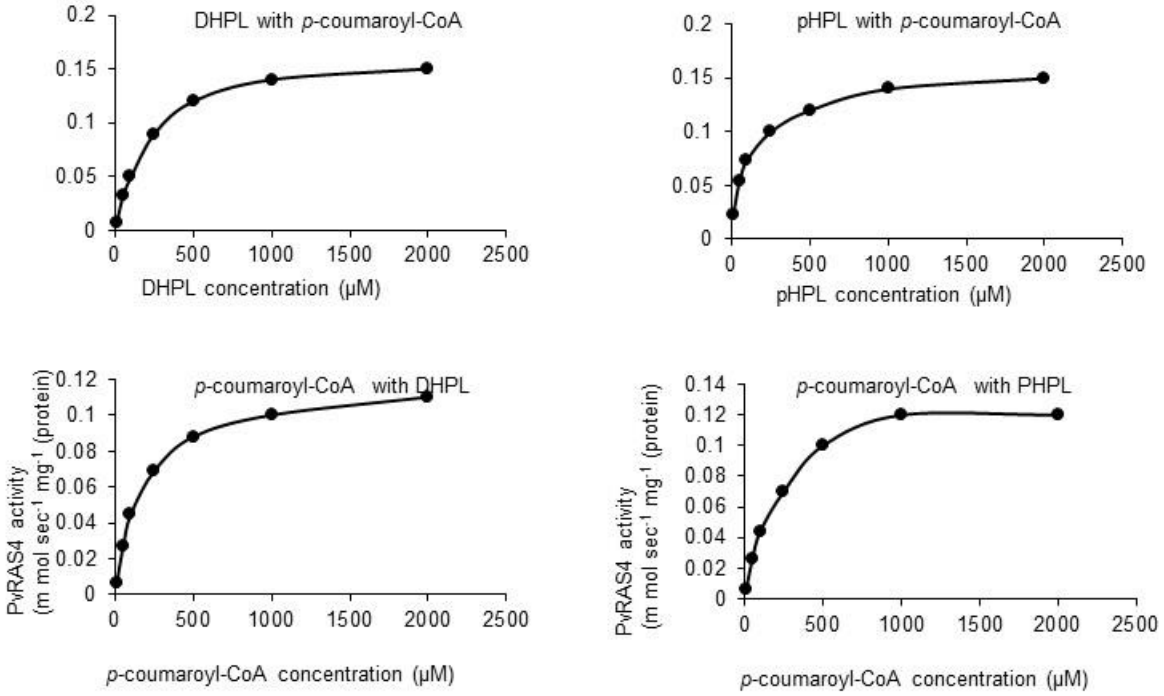

Fig. S13. Plots of initial velocity versus substrate concentration for recombinant PvRAS4 with donors and acceptors as substrates.

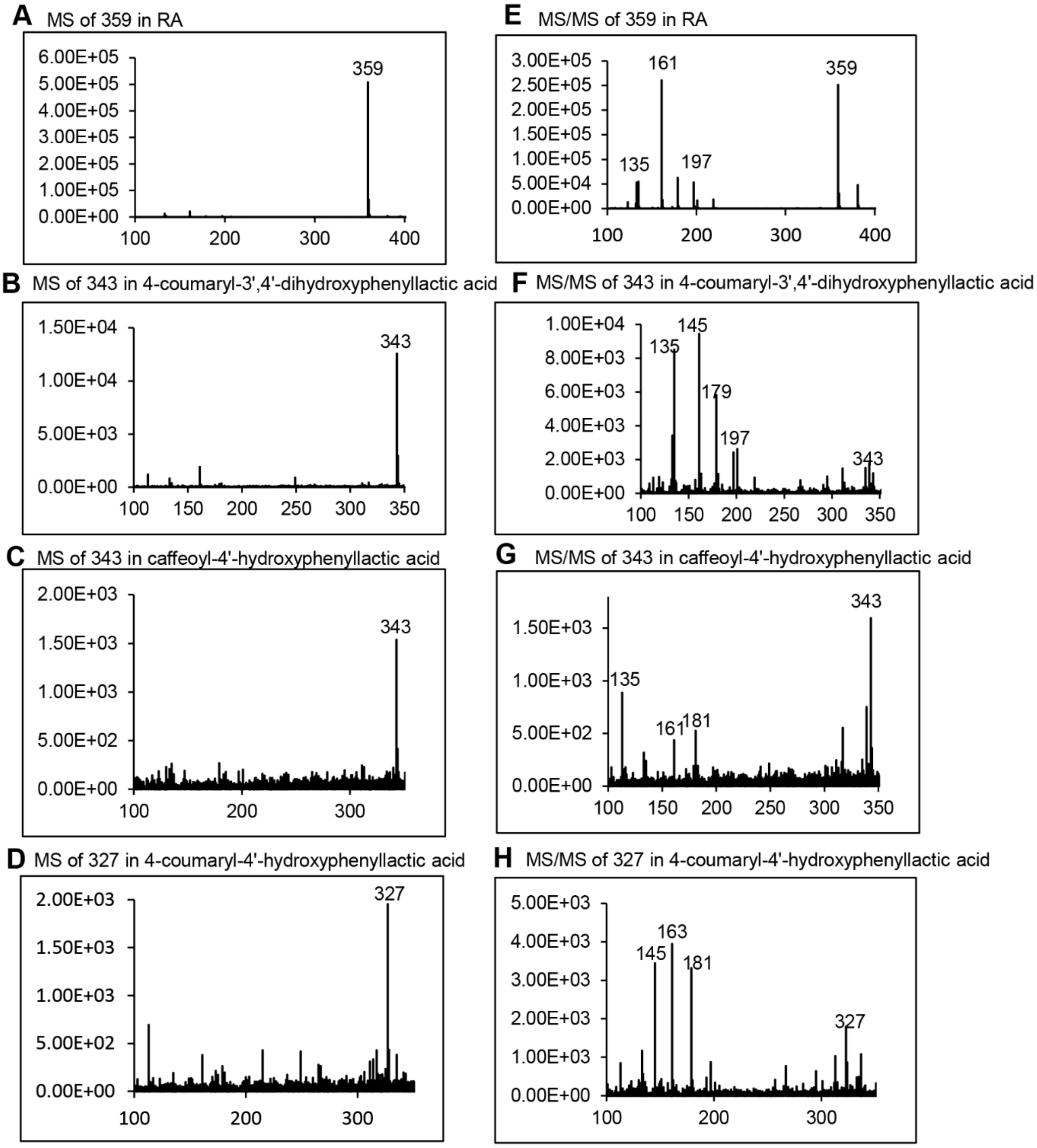

Fig. S14. MS/MS spectra of RA, 4-coumaroyl-3',4'-dihydroxyphenyllactic acid, 4-coumaroyl-4'-hydroxyphenyllactic acid, and caffeoyl-4'-hydroxyphenyllactic acid.
